# Endothelial-to-Osteoblast Conversion maintains bone homeostasis through Kindlin-2/Piezo1/TGFβ/Runx2 axis

**DOI:** 10.1093/procel/pwae066

**Published:** 2024-12-02

**Authors:** Guixing Ma, Yingying Han, Wanze Tang, Bo Zhou, Litong Chen, Zhen Ding, Siyuan Cheng, Di Chen, Huiling Cao

**Affiliations:** Department of Biochemistry, School of Medicine, Southern University of Science and Technology, Guangdong Provincial Key Laboratory of Cell Microenvironment and Disease Research, Shenzhen Key Laboratory of Cell Microenvironment, Key University Laboratory of Metabolism and Health of Guangdong, Southern University of Science and Technology, Shenzhen 518055, China; Department of Biochemistry, School of Medicine, Southern University of Science and Technology, Guangdong Provincial Key Laboratory of Cell Microenvironment and Disease Research, Shenzhen Key Laboratory of Cell Microenvironment, Key University Laboratory of Metabolism and Health of Guangdong, Southern University of Science and Technology, Shenzhen 518055, China; Department of Biochemistry, School of Medicine, Southern University of Science and Technology, Guangdong Provincial Key Laboratory of Cell Microenvironment and Disease Research, Shenzhen Key Laboratory of Cell Microenvironment, Key University Laboratory of Metabolism and Health of Guangdong, Southern University of Science and Technology, Shenzhen 518055, China; Department of Biochemistry, School of Medicine, Southern University of Science and Technology, Guangdong Provincial Key Laboratory of Cell Microenvironment and Disease Research, Shenzhen Key Laboratory of Cell Microenvironment, Key University Laboratory of Metabolism and Health of Guangdong, Southern University of Science and Technology, Shenzhen 518055, China; Department of Biochemistry, School of Medicine, Southern University of Science and Technology, Guangdong Provincial Key Laboratory of Cell Microenvironment and Disease Research, Shenzhen Key Laboratory of Cell Microenvironment, Key University Laboratory of Metabolism and Health of Guangdong, Southern University of Science and Technology, Shenzhen 518055, China; Department of Biochemistry, School of Medicine, Southern University of Science and Technology, Guangdong Provincial Key Laboratory of Cell Microenvironment and Disease Research, Shenzhen Key Laboratory of Cell Microenvironment, Key University Laboratory of Metabolism and Health of Guangdong, Southern University of Science and Technology, Shenzhen 518055, China; Department of Biochemistry, School of Medicine, Southern University of Science and Technology, Guangdong Provincial Key Laboratory of Cell Microenvironment and Disease Research, Shenzhen Key Laboratory of Cell Microenvironment, Key University Laboratory of Metabolism and Health of Guangdong, Southern University of Science and Technology, Shenzhen 518055, China; Research Center for Computer-aided Drug Discovery, Shenzhen Institute of Advanced Technology, Chinese Academy of Sciences, Shenzhen 518055, China; Faculty of Pharmaceutical Sciences, Shenzhen University of Advanced Technology, Shenzhen 518055, China; Department of Biochemistry, School of Medicine, Southern University of Science and Technology, Guangdong Provincial Key Laboratory of Cell Microenvironment and Disease Research, Shenzhen Key Laboratory of Cell Microenvironment, Key University Laboratory of Metabolism and Health of Guangdong, Southern University of Science and Technology, Shenzhen 518055, China

**Dear Editor,**

Angiogenesis, the process of forming new blood vessels, is closely linked to osteogenesis, the formation of new bone tissue, which is important for bone development and homeostasis (Kusumbe et al., 2014). Recent studies have established that Endothelial-to-Osteoblast Conversion (EC-to-OSB), mediated by Endothelial-to-Mesenchymal Transition and subsequent osteogenesis, plays a crucial role ectopic ossification during cancer bone metastasis (Lin et al., 2017; Medici et al., 2010). Moreover, research suggests that endothelial cells are one of the sources of bone marrow mesenchymal stem cells (BMSCs) which contribute to the reconstruction of the bone marrow niche during bone marrow transplantation (Kenswil et al., 2021). However, this view remains controversial, as other studies (Cao et al., 2023). Since it remains unclear whether and how EC-to-OSB regulates bone formation and homeostasis under physiological or aged conditions. Therefore, further research on the pathogenesis of osteoporosis (OP) is urgent.

EC-to-OSB plays an essential role in ectopic ossification during cancer bone metastasis. However, the exact physiological function of EC-to-OSB in regulating bone formation remains unclear. To address this issue, we analyzed our previous single-cell sequencing data (Fu et al., 2020) and identified a population of cells with high expression of endothelial cell marker genes in BMSC (Fig. S1). Based on the above findings, we hypothesized that endothelial cells may play an important role in bone homeostasis through EC-to-OSB. Since transforming growth factor β (TGFβ) plays an important role in enhancing EC-to-OSB (Medici et al., 2010), we further verified the hypothesis by using SB431542 (SB), a TGFβ inhibitor (Zhen et al., 2013) *in vivo* (Fig. S2A–C). Blocking EC-to-OSB using SB resulted in a decrease in bone mass revealed by microcomputed tomography (μCT) analysis (Fig. S2D–F). Interestingly, inhibition of TGFβ by SB (10 mg/kg for a month) primarily affected the osteogenic process, as demonstrated by decreased osteocalcin (Ocn), an osteogenic marker, revealed by immunofluorescent (IF) staining, and osteoclast formation was not significantly altered, as determined by tartrate-resistant acid phosphatase (Trap) staining (Fig. S2G–J). The decrease in osteoblasts may result from the blockage of EC-to-OSB, as evidenced by the reduced number of endothelial-derived osteoblasts (labeled by endothelial markers Tie2 or Cd31 and osteogenic markers Ocn or Runx2) on the trabecular bone surface (Fig. S2K–O). Runx2 is a master transcription factor controlling osteoblast differentiation (Takarada et al., 2016). we constructed a mouse model with *Runx2* gene deletion in endothelial cells (*Tie2*^*cre*^*; Runx2*^*fl/fl*^) to block the osteoblastic differentiation of endothelial cells. μCT analysis revealed that the bone mass of *Tie2*^*cre*^*; Runx2*^*fl/fl*^ and *Tie2*^*cre*^*; Runx2*^*fl/+*^ mice was similarly decreased (Fig. 1A–C). Further analysis confirmed that the bone mass loss was not caused by altered osteoclast formation but EC-to-OSB blockage (Figs. 1D–F and S2P–T).

**Figure 1.**
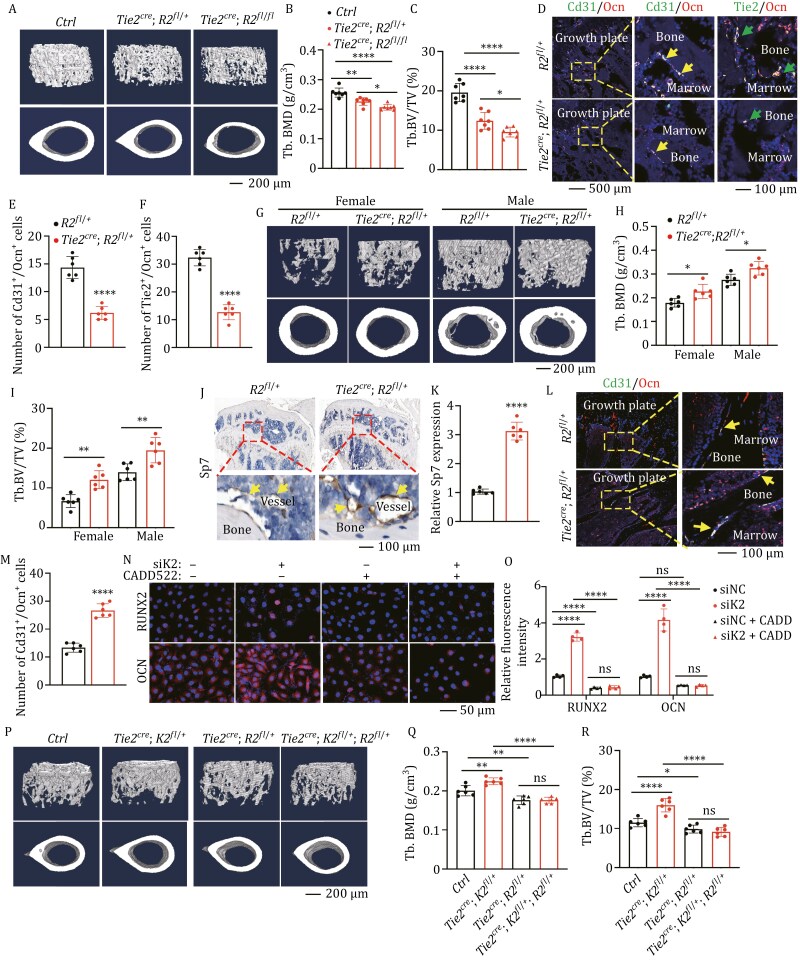
EC-to-OSB increases bone mass under physiological conditions through Kindlin-2. (A–C) 3D reconstruction (A) and quantitative analyses of BMD (B) and BV/TV (C) from μCT scans of the distal femurs of *Tie2*^*cre*^*; Runx2*^*fl/fl*^ mice and control mice. *n* = 6 mice for each group. Scale bar, 200 μm. (D–F) IF staining. Tibial sections of *Tie2*^*cre*^*; Runx2*^*fl/+*^ mice using indicated antibodies. Scale bars, 500 and 100 μm. The arrow indicated Ocn^+^ Cd31^+^ cells and Ocn^+^ Tie2^+^ cells. Female mice, *n* = 6 mice for each group. (G–I) 3D reconstruction (G) and quantitative analyses of BMD (H) and BV/TV (I) from μCT scans of the distal femurs of *Tie2*^*cre*^*; K2*^*fl/+*^ mice and control mice at 2 month of age. Scale bar, 200 μm. *n* = 6 mice for each group. (J and K) IHC staining of Sp7 and its quantitative analyses. Tibial sections of *Tie2*^*cre*^*; Runx2*^*fl/+*^ mice and control mice were subjected to IHC staining. *n* = 6 mice for each group. The yellow arrow indicated endothelial cells. (L and M) IF staining of endothelial derived osteoblasts and its quantitative analyses. Tibial sections of *Tie2*^*cre*^*; K2*^*fl/+*^ mice and control mice at 2 month of age were subjected to IF staining using antibodies against Ocn and Cd31. *n* = 6 mice for each group. The yellow arrow indicated Cd31^+^/Ocn^+^ cells. Scale bar, 100 μm. (N and O) IF staining if Ocn (N) and its quantitative analyses (O). (P–R) 3D reconstruction (P) and quantitative analyses of BMD (Q) and BV/TV (R) from μCT scans of the distal femurs of *Tie2*^*cre*^; *K2*^*fl*/+^; *Runx2*^*fl*/+^ and control mice. Female mice, *n* = 6 for each group. Scale bar, 1 mm. ^*^P < 0.05, ^**^P < 0.01, ^***^P < 0.001, ^****^P < 0.0001, versus controls. Student’s *t*-test for two group or one-way ANOVA for multiple groups. Results are expressed as the mean ± SD.

Since BMP4 and TGFβ stimulation have been shown to promote EC-to-OSB (Medici et al., 2010), we further investigated the signaling pathways involved in EC-to-OSB. We found that pathways related to cell adhesion and focal adhesion were significantly altered upon BMP4 or TGFβ stimulation (Fig. S3A and S3B). We hypothesized that Kindlin-2, a focal adhesion protein, might plays a role in EC-to-OSB–mediated bone homeostasis. Then, we knocked down KINDLIN-2 expression in human umbilical vein endothelial cell (HUVECs) and found that these cells underwent significant morphological changes, from an endothelioid to fibroblast-like phenotype, indicating enhanced mesenchymal-like transformation (Fig. S3C). Alizarin red staining revealed that HUVECs with KINDLIN-2 knockdown displayed significantly enhanced bone forming activity (Fig. S3C and S3D). These results together with the downregulation of VE-cadherin (VE-CAD) and upregulation of N-cadherin (N-CAD) and RUNX2 strongly indicate that Kindlin-2 knockdown dramatically promotes EC-to-OSB (Fig. S3E–G).

Furthermore, we developed an endothelial Kindlin-2 knockout mouse model. Since homozygous knockout of Kindlin-2 was fatal at embryonic day (E) 10.5 (Dong et al., 2023b), we used *Tie2*^*cre*^*; Kindlin-2*^*fl/+*^ mice (*Tie2*^*cre*^*; K2*^*fl/+*^) for further study (Fig. S4A and S4B). *Tie2*^*cre*^*; K2*^*fl/+*^ mice showed no observable abnormalities and a similar body weight compared with control mice but increased bone mass without affecting structure of vessels and osteoclast formation (Figs. 1G–Iand S4C–F). Calcein blue double-labeling, Von Kossa staining, and IF staining of Ocn, a marker of mature osteoblast, all suggested that the bone mass increase in *Tie2*^*cre*^*; K2*^*fl/+*^ mice was mainly due to enhanced bone formation (Fig. S4G–N). In addition, SP7, a marker of pre-osteoblasts, was significantly increased in endothelial cells (Fig. 1J–K). The number of endothelial-derived (Cd31^+^/Ocn^+^) osteoblasts on trabecular bone surface in *Tie2*^*cre*^*; K2*^*fl/+*^ mice was significantly increased (Fig. 1L and 1M). Further experiments using the RUNX2 inhibitor CADD522 abolished the increased expression of SP7 or OCN caused by KINDLIN-2 knockdown in HUVECs (Figs. 1N, 1O, S4O and S4P). μCT analysis revealed that the high bone mass phenotype of *Tie2*^*cre*^*; Kindlin-2*^*fl/+*^ mice was reversed by Runx2 haploinsufficiency through inhibition of EC-to-OSB (Figs. 1P–R and S4Q–S). Collectively, these findings indicate that the increase in bone mass caused by endothelial Kindlin-2 haploinsufficiency is mediated through Runx2-dependent EC-to-OSB.

RNA sequencing analysis of HUVECs after RNAi treatment revealed that Kindlin-2 knockdown resulted in the activation of endothelial cell proliferation and osteoblast differentiation pathways (Fig. S5A). This was further evidenced by IF staining (Fig. S5B and S5C). Further results showed that the mRNA levels of BMPs were slightly decreased, while TGFβ1 and TGFβ2 were significantly increased following KINDLIN-2 knockdown in HUVECs (Fig. S5D–J; Table S1–3). Results of tibial sections confirmed the elevated TGFβ levels in endothelial cells and osteoblasts in *Tie2*^*cre*^*; K2*^*fl/+*^ mice (Fig. S5K–M). The increase in cell proliferation caused by KINDLIN-2 knockdown was abolished by SB treatment (Fig. S6A and S6B), consistent with TGFβ’s known ability to promote cell proliferation (Zhen et al., 2013). Additionally, the increased RUNX2 expression resulting from KINDLIN-2 knockdown was reversed by SB treatment both *in vivo* and *in vitro* (Fig. S6C–L). These data suggest that it is TGFβ pathway functions downstream of Kindlin-2 in modulating EC-to-OSB.

RNA sequencing results showed that KINDLIN-2 knockdown significantly affected the calcium signaling pathway (Table S4). Furthermore, knocking down KINDLIN-2 significantly increased Ca^2+^ level and NFATc1 protein level in HUVECs (Fig. S7A–D). KN-93, a calcium signaling antagonists, was able to reverse the increased NFATc1 (Fig. S7E and S7F). Additionally, NFATc1 was also significantly increased in bone tissues of *Tie2*^*cre*^*; K2*^*fl/+*^ mice (Fig. S7G–I). Piezo1 is a key calcium channel that senses fluid shear stress (Li et al., 2014). Here, we found that KINDLIN-2 knockdown significantly increased PIEZO1 expression, while KINDLIN-2 overexpression dramatically reduced it (Figs 2A, 2B, and S7J-N). IHC staining confirmed the increase of Piezo1 in mice (Fig. S7O and S7P). We next treated HUVECs with Yoda1, a Piezo1 agonist (Syeda et al., 2015), and found that Yoda1-induced Piezo1 activation significantly promoted EC-to-OSB and increased expression of TGFβ, pSMAD2/3, RUNX2, and SP7 (Fig. S7Q–T). While treated with GsMT×4, an unspecific ion channel antagonist which also antagonizes Piezo1 activity (Bae et al., 2011), we found that the increase of PIEZO1, TGFβ, and RUNX2 was abolished (Figs. 2C, 2D, S8A and S8B).

**Figure 2.**
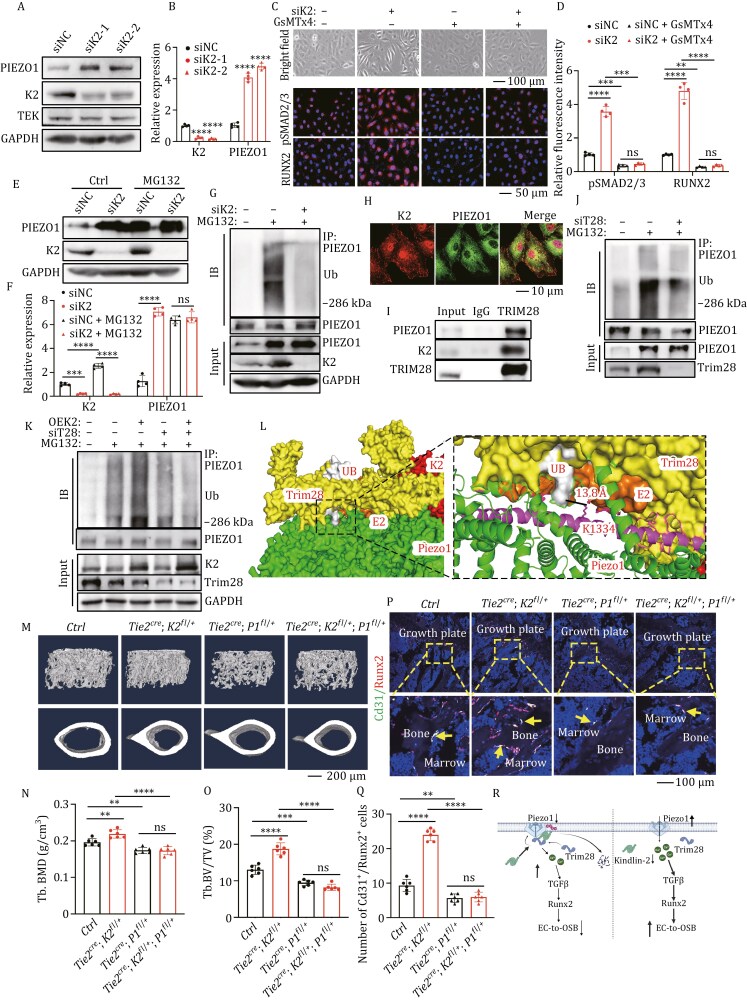
Kindlin-2 promotes Piezo1 protein degradation through enhancing Trim28-dependent ubiquitination. (A and B) WB analyses and quantitative analyses of HUVECs after KINDLIN-2 were knocked down. (C and D) Bright field, IF staining (C) and quantitative analyses (D) of HUVECs with KINDLIN-2 knockdown followed by GsMT×4 treatment. Scale bar, 50 μm. (E and F) WB and quantitative analyses. Protein extracts were isolated from HUVECs after MG132 treatment and KINDLIN-2 knockdown. *n* = 4 biologically independent experiments. (G) WB analyses for Piezo1 ubiquitination. Protein extracts were isolated from HUVECs after MG132 treatment and KINDLIN-2 knockdown. (H) IF staining for detecting the colocalization of KINDLIN-2 and PIEZO1 in HUVECs. Scale bar, 10 μm. (I) Co-IP assays. Cell lysates from HUVECs were used for IP and IB assays for detecting the endogenous interaction between TRIM28 and KINDLIN-2, and TRIM28 and PIEZO1. (J) WB analyses of PIEZO1 ubiquitination. Protein extracts were isolated from HUVECs after MG132 treatment and TRIM28 knockdown. (K) WB analyses for PIEZO1 ubiquitination. Protein extracts were isolated from HUVECs after MG132 treatment, KINDLIN-2 overexpression, and TRIM28 knockdown. (L) Protein structure of the Kindlin-2-Piezo1-Trim28-E2 ligase complex predicted by AlphaFold2 Swiss-Model and HDOCK, and photographs were displayed by Pymol and Chimera. (M–O) 3D reconstruction (M) and quantitative analyses of BMD (N) and BV/TV (O) from μCT scans of the distal femurs from *Tie2*^*cre*^; *K2*^*fl*/+^; *Peizo1*^*fl*/+^ and control mice. Female mice, *n* = 6 mice per group. (P and Q) IF staining of endothelial derived osteoblasts and its quantitative analyses. Tibial sections of *Tie2*^*cre*^; *K2*^*fl*/+^; *Piezo1*^*fl*/+^ and control mice were subjected to IF staining using indicated antibodies. The yellow arrow indicated Cd31^+^/Runx2^+^ cells on trabecular surface. Scale bar, 100 μm. (R) Working model of Kindlin-2 regulation of Piezo1 protein stability. ^*^P < 0.05, ^**^P < 0.01, ^***^P < 0.001, ^****^P < 0.0001, versus controls, Student’s *t*-test, or one-way ANOVA. Results are expressed as the mean ± SD.

Furthermore, we determined the mechanisms by which Kindlin-2 regulates Piezo1. Results of qRT-PCR analysis, Cycloheximide (CHX), a compound that inhibits protein synthesis, and MG132, a proteasome inhibitor that blocks protein degradation, experiments demonstrated that Kindlin-2 deficiency increased Piezo1, mainly through inhibition of proteasome-mediated protein degradation (Figs. 2E–G and S8C–E). IF staining and co-immunoprecipitation (Co-IP) experiments demonstrated a strong interaction between KINDLIN-2 and PIEZO1 (Figs. 2H, S8F and S8G). Further mass spectrometric analysis on Kindlin-2 Co-IP samples identified Trim28, an E3 ligase (Dong et al., 2023a), as a potential mediator (Table S5; Figs. 2I, S8H and S8I). Knockdown of KINDLIN-2 had no effect on TRIM28 protein level (Fig. S8J and S8K), and knockdown of TRIM28 inhibited the ubiquitination of PIEZO1 (Fig. 2J). While KINDLIN-2 knockdown reduced the colocalization of PIEZO1 and TRIM28 (Fig. S8L), and TRIM28 knockdown effectively abolished Kindlin-2 overexpression-induced increase of Piezo1 ubiquitination (Fig. 2K).

Using structure prediction by AlphaFold-multimer and Swiss-Model, followed by molecular docking using HDOCK (Fig. S9), we revealed that the Ring domain of the Trim28 dimer interacted with the Piezo1 trimer, along with E2 and UB, where UB molecule pointed toward the Beam Domain near pivot position of Piezo1 (Fig. 2L). The distance between the Gly residue at the UB C terminus and Piezo1 K1334, which was validated by different mass spectrometry data (Beltrao et al., 2012), is 13.8 Å (Fig. 2L). Additionally, Kindlin-2 interacted with the Trim28 dimer through the F0 domain at the opposite end of Trim28 dimer (Fig. S10E). While Piezo1 interacted with the PH domain of Kindlin-2 (Fig. S10E), we validated the above hypothesis using plasmids expressing different truncations of Kindlin-2 through Co-IP. The results showed that Kindlin-2^aa1-239^ interacted with Trim28, while its interaction with Piezo1 required the integrity of Kindlin-2^aa1-569^ (Fig. S8M).

To further verify whether Kindlin-2 knockdown can exert its effect through Piezo1 *in vivo*, given that the impact of endothelial cell Piezo1 on osteogenesis is still unclear, we crossed *Tie2*^*cre*^ mice with *Piezo1*^*fl/fl*^ mice to obtain mice with one allele of *Piezo1* gene deletion in endothelial cells (*Tie2*^*cre*^*; Piezo1*^*fl/+*^) (Fig. S10A–C), since the homozygous mice are lethal (Li et al., 2014). The significant decrease in bone mass in *Tie2*^*cre*^*; Piezo1*^*fl/+*^ mice, and the unaffected osteoclast formation, together with the decrease in Cd31^+^ osteoblasts, demonstrated the crucial role of endothelial Piezo1 in maintaining bone homeostasis through promoting EC-to-OSB (Fig. S10F–K). Next, we generated *Kindlin-2* and *Piezo1* genes double heterozygous mice using *Tie2*^*cre*^ mice (*Tie2*^*cre*^*; K2*^*fl/+*^*; P1*^*fl/+*^ mice) and found Piezo1 haploinsufficiency successfully reversed the high bone mass phenotype of *Tie2*^*cre*^*; K2*^*fl/+*^ mice (Fig. 2M–Q).

Collectively, the above results suggest that Kindlin-2 functions as a linker for the interaction between Trim28 and Piezo1, enhancing Trim28-dependent Piezo1 ubiquitination and degradation, ultimately inhibiting EC-to-OSB and decreases bone mass (Fig. 2R).

OP, commonly observed in postmenopausal women and the elderly, is considered as an age-related othorpaedic disease. Results from ovariectomized (OVX) and aged mice (18-month-old) showed a notable reduction in Cd31^+^/Runx2^+^ osteoblasts on trabecular bone surface (Fig. S11A–D), and a substantial decrease in Piezo1 and increase in Kindlin-2 expression in Cd31^+^ osteoblasts during aging observed both in mice and humans with OP (Fig. S11E and S11M).

To explore the potential translational significance of targeting endothelial Kindlin-2 in OP treatment, and to exclude the non-vascular activation of Tie2-Cre during development (Cao et al., 2023), we employed CasRx-mediated mRNA editing technology in mice. Specifically, sgRNAs targeting *Kindlin-2* demonstrated by our previous work were utilized (Tang et al., 2023), and CasRx was placed under the control of the *CDH5* promoter and packaged into adeno-associated viruses (AAVs) (Fig. S11N). μCT analysis revealed a significant increase in bone mass in sgK2-treated aged mice through an increased EC-to-OSB (Fig. S11O–V). Additionally, we established an OVX mouse model to investigate the therapeutic effects of sgK2-AAVs in this context. As shown in Fig. S11W–Y, endothelial Kindlin-2 knockdown effectively protected against OVX-induced OP.

Collectively, these findings highlight the potential role of EC-to-OSB and Kindlin-2/Piezo1/TGFβ/Runx2 axis in the maintenance of bone homeostasis and CasRx-mediated endothelial *Kindlin-2* mRNA editing represents a promising therapeutic strategy for the treatment of age-related OP.

## Supplementary data

Supplementary data is available at *Protein & Cell* online https://doi.org/10.1093/procel/pwae066.

pwae066_suppl_Supplementary_Material

## Footnotes

We acknowledge the assistance of Core Research Facilities of the Southern University of Science and Technology. This work was supported, in part, by the National Key Research and Development Program of China Grants (2019YFA0906001), the National Natural Science Foundation of China Grants (82372476 and 82350710800), and Guangdong Provincial Science and Technology Innovation Council Grant (2017B030301018). The authors declare that they have no competing interests.

Study design: Guixing Ma, Yingying Han, Di Chen, and Huiling Cao. Study conducts, data collection, and analysis: Guixing Ma, Yingying Han, Wanze Tang, Bo Zhou, Zhen Ding, Litong Chen, Siyuan Cheng, and Huiling Cao. Data interpretation: Huiling Cao, Guixing Ma, and Yingying Han. Drafting the manuscript: Huiling Cao and Guixing Ma. Di Chen and Huiling Cao take the responsibility for the integrity of the data analysis.

This study was approved by Ethics Committee of the hospital (IRB No: 2021-192). All institutional and national guidelines for the care were carefully followed. Additional informed consent was obtained from all patients for which identifying information is included in this article. All institutional and national guidelines for the care and use of laboratory animals were followed.
