# Correction to: Endothelial-to-Osteoblast Conversion maintains bone homeostasis through Kindlin-2/Piezo1/TGFβ/Runx2 axis

**DOI:** 10.1093/procel/pwaf056

**Published:** 2025-08-09

This is a correction to: Guixing Ma, Yingying Han, Wanze Tang, Bo Zhou, Litong Chen, Zhen Ding, Siyuan Cheng, Di Chen, Huiling Cao, Endothelial-to-Osteoblast Conversion maintains bone homeostasis through Kindlin-2/Piezo1/TGFβ/Runx2 axis, *Protein & Cell*, Volume 16, Issue 6, June 2025, Pages 497–502, https://doi.org/10.1093/procel/pwae066

In the originally published version of this manuscript, corrections were required to Figs. 2, S3 and S7.

In Fig. 2M–Q, the group labels were incorrect. The correct labels should be: *Ctrl, Tie2^*cre*^; K2^*fl/+*^, Tie2^*cre*^; P1*^*fl/+*^, and *Tie2^*cre*^; K2^*fl/+*^ P1^fl/+^*. They were mistakenly labeled as *Ctrl, Tie2^cre^; K2*fl/+*, Tie2^*cre*^; R2^*fl/+*^, and Tie2^*cre*^; K2^*fl/+*^ R2^*fl/+*^*.

In Fig. S3, there was an extra letter before “Kindlin-2” in the legend. The updated version has removed this.

In Fig. S7R, the labels should be *Ctrl* and *Yoda1*, which were previously incorrectly shown as *siNC* and *siK2*.

These corrections have been made in the online version of the article.

