# Supplementary material for: Endothelial-to-Osteoblast Conversion maintains bone homeostasis through Kindlin-2/Piezo1/TGFβ/Runx2 axis
Source: Protein Cell. 2024 Dec 2;16(6):497–502. doi: 10.1093/procel/pwae066 (PMC12187329; doi:10.1093/procel/pwae066)
Supplement: pwae066_suppl_Supplementary_Material [file pwae066_suppl_supplementary_material.pdf]

Supplementary information

Endothelial-to-Osteoblast Conversion maintains bone homeostasis through Kindlin-2/Piezo1/TGFβ/Runx2 axis

Guixing Ma[1#], Yingying Han[1#], Wanze Tang[1a], Bo Zhou[1], Litong Chen[1], Zhen Ding[1],

Siyuan Cheng[1], Di Chen[2*], Huiling Cao[1*]

This file includes:

Materials and methods

Tables S1-S5

Figures S1-S11

Materials and methods

Animal studies

Kindlin-2$^{fl/fl}$ mice(Wu et al., 2015), *Piezo1*$^{fl/fl}$ mice(Cahalan et al., 2015) and *Runx2*$^{fl/fl}$ mice (Takarada et al., 2013) were described in previous studies. To generate endothelial specific knockdown of Kindlin-2, *Tie2*^{Cre}*; Kindlin-2*$^{fl/+}$ mice (referred to as *Tie2*^{Cre}*; K2*$^{fl/+}$ hereafter), endothelial specific knockdown of Runx2, *Tie2*^{Cre}*; Runx2*$^{fl/+}$ mice (referred to as *Tie2*^{Cre}*; R2*$^{fl/+}$) or endothelial specific knockdown of Piezo1, *Tie2*^{Cre}*; Piezo1*$^{fl/+}$ mice (referred to as *Tie2*^{Cre}*; P1*$^{fl/+}$), *Tie2*^{Cre} transgenic mice(Kisanuki et al., 2001) with Cre expression driven by the *Tie2* promoter were crossed with *Kindlin-2*$^{fl/fl}$ mice, *Runx2*$^{fl/fl}$ mice, *Piezo1*$^{fl/fl}$ mice, respectively. The Cre negative *Kindlin-2*$^{fl/+}$*, Runx2*$^{fl/+}$*, Piezo1*$^{fl/+}$ littermates were used as control (referred to as Ctrl) mice in this study. To generate endothelial specific knockdown of Kindlin-2 and Piezo1, *Tie2*^{Cre}*; Kindlin-2*$^{fl/+}$*; Piezo1*$^{fl/+}$ (referred to as *Tie2*^{Cre}*; K2*$^{fl/+}$*; P1*$^{fl/+}$) or endothelial specific knockdown of Kindlin-2 and Runx2, *Tie2*^{Cre}*; Kindlin-2*$^{fl/+}$*; Runx2*$^{fl/+}$ mice (referred to as *Tie2*^{Cre}*; K2*$^{fl/+}$*; R2*$^{fl/+}$), *Tie2*^{Cre}*; Kindlin-2*$^{fl/+}$ mice were crossed with *Piezo1*$^{fl/fl}$ mice or *Runx2*$^{fl/fl}$ mice, respectively. To block TGFβ signaling, SB431542(Li et al., 2015; Guan et al., 2022) (10 mg/kg) were intraperitoneally injected into 8 weeks old mice once a day for 2 months, respectively. Mice in the control group were injected with the solvent for SB431542. OVX mice were as previously described (Fu et al., 2020). 18-month-old mice were considered aged mice in this study. Mice were caged at the density of 5 mice/cage with free access to food and water. Room temperature was maintained between 20 to 24°C. All mice were maintained in C57BL/6 background. All animal experiments conducted in this study received approval from the Institutional Animal Care and Use Committee (IACUC) at the University. We confirm our adherence to all pertinent ethical regulations concerning animal testing and research in this study.

AAVs injection

CasRx mediated *Kindlin-2* mRNA editing was designed and performed as previously described(Tang et al., 2023). Adeno-associated virus serotype 9 (AAV9) carrying a CasRx gene under *CDH5* promoter and sgRNA4 under *U6* promoter. For *in vivo* study, 18-month old WT C57BL/6 mice were administrated with AAVs via tail vein injection at a dose of 10^{10} vg/mouse in 150 μL dilution buffer. After 2 months of injection, mice were sacrificed for further evaluation. 2-month old female mice are used to establish an ovariectomy (OVX) model, and two weeks post-OVX, AAVs are administered via tail vein injection at a dose of 10^{10} vg/mouse in 150 μL dilution buffer. After 2 months of injection, mice were sacrificed for further evaluation.

Micro-computerized tomography (μCT) analysis

Fixed femurs were used for μCT analysis as previously described(Bouxsein et al., 2010), using a Bruker μCT (SkyScan 1172 Micro-CT, Bruker MicroCT) following the standards of techniques and terminology suggested by the American Society for Bone and Mineral Research. Briefly, non-demineralized femurs were scanned at 60 KV, 100 μA, 926 ms with a 10 μm voxel size. For trabecular measurements, a region of interest of 1.0 mm length of distal femur was analyzed. For cortical bone analysis, a region of interest of 1.0 mm length of mid-femoral cortical bone was analyzed. The analyses of the specimens involved the following bone measurements: the BMD, BV/TV.

Bone histological evaluation

Tibias were fixed in 4% PFA overnight at 4 °C, followed by decalcification in 10% EDTA for 3 weeks as previously described(Wu et al., 2015). After decalcification, bones were embedded in paraffin and sectioned at a thickness of 5 μm, then deparaffinized, rehydrated, and utilized for HE staining. Stain with hematoxylin for 5-10 minutes to color the nuclei. Rinse in running tap water and differentiate in acid alcohol if necessary. Blue the sections in alkaline water or ammonia

solution.Counterstain with eosin for 1-2 minutes. Dehydrate sections through ethanol, clear in xylene, and mount with a coverslip using a synthetic resin.

***In vitro* osteoblastic differentiation**

In vitro osteoblastic differentiation of HUVECs were as previously described(Medici et al., 2010; Lin et al., 2017). HUVECs were seeded in 6-well plate at a density of 5×10^5 cells/well and cultured in osteogenic medium (α-MEM containing 10% FBS and 50 μg/ml ascorbic acid) for 7 days followed by qPCR analysis or changed to mineralization inducing medium (osteogenic medium plus 2.5 mM β-glycerophosphate) for another 7-14 days, followed by alizarin red staining (40 mM, pH 4.2)(Medici et al., 2010; Yan et al., 2022).

Osteoclast assays

Tibial sections were used for TRAP staining for evaluation of *in vivo* osteoclast formation and was performed as we previously described(Yan et al., 2022). For TRAP staining, incubate tissue sections in TRAP staining solution at 37°C for 30-60 minutes. Rinse in tap water followed by distilled water. Counterstain with a light green or hematoxylin counterstain, if desired. Rinse, dehydrate through increasing ethanol concentrations, clear in xylene, and apply coverslips using a mounting medium. BMMs were used for the *in vitro* osteoclast differentiation assay as we previously described(Yan et al., 2022). Non-adherent BMMs were isolated after culturing total bone marrow cells in tissue culture dishes for 48 hours. To induce differentiation, cells were first cultured in proliferation medium (α-MEM with 10% FBS and 10 ng/mL human recombinant M-CSF) for 3 days, followed by differentiation medium (proliferation medium plus 50 ng/mL human recombinant RANKL) for 4-7 days. TRAP-positive multinucleated cells were then assessed using an inverted microscope.

siRNA and plasmids

The negative control and siRNAs used in this study were synthesized by Suzhou GenePharma Co., Ltd (Suzhou, China). The Flag-Kindlin-2 and its truncation constructs were as previously described(Gao et al., 2022).

Von Kossa assay

Von Kossa assay was performed as previously described (Aikawa et al., 2007). Sections were fixed in 10% formalin for 15 minutes, then rinsed in distilled water. Slides were incubated in 5% silver nitrate solution under UV light for 30 minutes. After washing with distilled water, slides were treated with 5% sodium thiosulfate for 5 minutes to remove unreacted silver. Sections were then rinsed in distilled water and counterstained with nuclear fast red for 5 minutes. After a final rinse, slides were dehydrated through graded ethanol and cleared in xylene before mounting.

Cell culture and transfection

HUVECs were cultured in DMEM/F12 containing 10% FBS. Transient transfection was conducted when cells reached to 80% confluence with specific siRNAs using lipofectamine RNAiMAX transfection reagent (Invitrogen, cat# 13778150) and plasmids using lipofectamine 3000 transfection reagent (Invitrogen, cat# L3000015) according to the manufacturers' protocols as we previously described (Yan et al., 2022).

EdU assay

Cells were cultured on coverslips and incubated with 10 μM EdU for 2 hours. After incubation, cells were fixed in 4% paraformaldehyde for 15 minutes and washed with PBS. Cells were permeabilized with 0.5% Triton X-100 for 20 minutes and rinsed again with PBS. EdU incorporation was detected by incubating cells with a Click-iT reaction cocktail for 30 minutes in the dark. After washing with PBS, cells were counterstained with DAPI for 5 minutes to visualize nuclei. Coverslips were then mounted onto slides using antifade mounting medium.

Cytosol Ca^{2+} assay

The Ca^{2+} sensitive fluorescent dye Fluo-2/AM (Beyotime) was used to detect cytoplasmic Ca^{2+} levels. Briefly, HUVECs seeded in confocal dish were washed twice with D-Hank's buffer and incubated with 5 μM Fluo-2/AM at 37°C for 30 min away from light. Then, the cells were washed three times with D-Hank's buffer and further incubated with D-Hank's buffer at 37°C for another 30 min. Finally, signals were collected with a confocal laser scanning microscope (Nikon Confocal A1R system) using a 20x objective lens at 488 nm. The cytoplasmic Ca^{2+} concentrations were recorded as total fluorescence intensity. The fluorescence intensity variation was recorded from cells (Yan et al., 2022).

Real-time quantitative reverse transcription PCR (qRT-PCR) analyses

RNA isolation, reverse transcription, and qRT-PCR analyses were conducted as previously described(Fu et al., 2020). Quantitative real-time RT-PCR analysis was performed to measure relative mRNA levels using SYBR Green kit (Bio-Rad Laboratories Inc.). Samples were normalized to Gapdh expression.

Western blot (WB) analyses

Protein isolation and Western blot analyses were performed as we previously described(Fu et al., 2020). Cells were lysed in RIPA buffer (Sigma, R0278), and protein concentration was determined using a BCA kit (Cwbio, CW0014). Proteins were separated by SDS-PAGE and transferred onto a PVDF membrane. Primary antibodies were incubated at 4 ° C for 12 hours, followed by HRP-conjugated secondary antibodies. Detection was performed using a chemiluminescent HRP substrate.

Immunohistochemistry (IHC) staining

IHC staining was performed on 5-μm paraffin sections of the decalcified tibiae with the indicated antibodies using the Envision System-HRP (DAB) kit (DAKO North America, Inc.) following the standard protocols as described previously (Yan et al., 2022). Briefly, slides were baked at 55° C for 45 minutes, deparaffinized in xylene, and rehydrated through a graded ethanol series. Antigen retrieval was done using 0.1% trypsin at 37° C for 10 minutes. Endogenous peroxidases were blocked with 3% H2O2 in PBS for 10 minutes. Sections were blocked for 30 minutes, incubated with the primary antibody at 4° C for 2 hours, and then washed. A donkey anti-rabbit IgG-HRP secondary antibody was applied for 30 minutes. HRP activity was visualized with DAB for 5 minutes, and sections were counterstained with Mayer's hematoxylin.

Immunofluorescence (IF) staining

Immunofluorescence staining was performed as previously described(Yan et al., 2022) on 5 μm paraffin sections of the decalcified tibiae. Briefly, the sections were dewaxed with xylene and rehydrated with 100% ethyl alcohol, 95% ethyl alcohol, 70% ethyl alcohol, and PBS. Sections were then performed antigen retrieval with citrate buffer (pH6.8) and incubated with immunostaining permeabilization solution with saponin (Beyotime, cat# P0095) for 10 min, and blocked with immunol staining blocking buffer (Beyotime, cat# P0102) for 1 h at room temperature. Following that, the primary antibody was diluted into the primary antibody dilution buffer (Beyotime, cat# P0262), and the sections were incubated with the diluted primary antibody for overnight at 4C°. Sections were washed with 1x PBS for 3 times and incubated with goat anti-rabbit alexa fluor 488 secondary antibody (Invitrogen, cat# A11008) diluted by secondary antibody dilution buffer (Beyotime, cat# P0265) at room temperature for 1 h. The sections were washed with 1 × PBS for 3 times and immediately mounted with mounting medium with DAPI (Abcam, cat# ab104139). The IF signal was acquired with Nikon Confocal A1R system under 20x objective lens.

Co-immunoprecipitation (Co-IP) assay

The co-immunoprecipitation (Co-IP) assay was conducted according to previously established protocols(Zhu et al., 2020). Briefly, the cells were subjected to a 10-minute incubation at 4 °C in an IP lysis buffer (pH 7.4, 0.025 M Tris, 0.15 M NaCl, 0.001 M EDTA, 1% NP-40, and 5% Glycerol) (Thermo Fisher), supplemented with a proteinase inhibitor cocktail. Subsequently, the lysate was centrifuged at 13,000 × g for 10 minutes at 4 °C, and the resulting supernatant was incubated overnight with the appropriate primary antibody, followed by incubation with Protein A/G Magnetic Beads at room temperature for 1 hour. The dynabeads-antigen-antibody complex was collected using a DynaMag™-2 Magnet (Thermo Fisher). The complex was then subjected to three washes with IP buffer, resuspended in 60 μl of 1× loading buffer, and heated at 95 °C for 5 minutes. Finally, the samples were separated by SDS-PAGE and subjected to Western blotting.

LC-MS/MS protein identification

Kindlin-2 Co-IP samples prepared using anti-Kindlin-2 antibody on 293T cells and analyzed using mass spectrometry. After Co-IP, the sample was subjected to enzymatic digestion using trypsin, resulting in the generation of peptide fragments from the proteins. Liquid chromatography-tandem mass spectrometry (LC-MS) was employed to analyze each peptide sample. The acquired spectrum files were evaluated using PD/MASCOT software to identify the proteins and E3 ligase listed in Table S5.

Single cell and RNA sequencing data analysis

To investigate the landscape of stromal cells in bone marrow, we re-analyzed published dataset of BMSC from our previous study (Fu et al., 2020) (GSE150291). Here, mesenchymal stromal cells (MSCs) were divided into 7 sub-populations, and vascular endothelial cells (VECs) were grouped into 5 sub-populations, according to their molecular signatures and showed by Uniform Manifold Approximation and Projection

(UMAP)(Becht et al., 2018). To identify their relationships, we adopted the expression levels of highly expressed genes for Pearson's correlation analysis. In brief, raw expression counts of UMIs for each sub-population were gathered as pseudo-bulk. And non-ribosomal and non-mitochondrial genes with pseudo-bulk higher than 2 were accepted for correlation analysis. The highly expressed genes in both $Lepr^+$ MSC and $Vcam1^+$ cVEC were employed for functional enrichment analysis with Metascape(Zhou et al., 2019). To investigate the pathways influenced by BMP4 and TGFβ, publicly available data (GSE70207, GSE168321) were utilized for functional enrichment analysis using Metascape(Zhou et al., 2019).

Structure prediction

For Piezo1/Trim28/Kindlin2 complex structure, we first utilized AlphaFold2(Jumper et al., 2021) and AlphaFold-multimer to predict the protein structures of Piezo1 monomer, Trim28 dimer, and Kindlin-2 monomer. The Piezo1 trimer structure was modeled using Swiss-Model(Waterhouse et al., 2018). Subsequently, the Trim28/E2/UB complex was manually docked using homology-based alignment by Pymol(Liang et al., 2003). The Piezo1 trimer, Trim28/E2/UB complex, and Kindlin-2 were further subjected to docking in HDOCK(Yan et al., 2017), eliminating unreasonable structures, to obtain the Piezo1/Trim28/Kindlin2 complex structure. The structure details were displayed by Pymol(Liang et al., 2003; Yuan et al., 2016) and Chimera (Pettersen et al., 2004).

Human samples

Human samples were collected from control individuals and patients with OP at Hospital, following approved procedures by the ethics committee of the hospital (IRB No: 2021-192). All participants provided written informed consent. Bone mineral density was measured, and individuals with T scores $\leq$ -2.5 were assigned to the OP group, while those with T scores $\geq$ -1 were assigned to the control group(Cummings et

al., 2009) (Table S3). Participants with other metabolic diseases such as diabetes, arthritis, osteonecrosis, etc., were excluded from the study.

Statistics

Mice used in experiments of this study were randomly grouped. IF, IHC, and histology were conducted and analyzed in a double-blinded way. Statistical analyses were performed using the GraphPad Prism 8. The two-tailed unpaired Student's t test (two groups) and two-way ANOVA (multiple groups), followed by Tukey's post-hoc test, were used. Results are expressed as mean ± standard deviation (sd), as indicated in the Figure Legends. Differences with $p < 0.05$ were considered of statistical significance.

Supplementary Tables

Table S1. Primer information used in this study.

Gene	Forward	Reverse
Kindlin-2	CAGACACCCCGAAGAACTTTC	GCCCCTCTAATTCAAGTGCCT
Piezo1	GGACTCTCGCTGGTCTACCT	GGGCACAATATGCAGGCAGA
TGFα	AGGTCCGAAAACACTGTGAGT	AGCAAGCGGTTCTTCCCTTC
TGFβ1	GGCCAGATCCTGTCCAAGC	GTGGGTTTCCACCATTAGCAC
TGFβ2	CAGCACACTCGATATGGACCA	CCTCGGGCTCAGGATAGTCT
TGFβ3	ACTTGCACCACCTTGGACTTC	GGTCATCACCGTTGGCTCA
BMP1	GGACGGGATAAGGATGCCA	CATCGAGTTTTTCCACCAAC
BMP2	ACCCGCTGTCTTCTAGCGT	TTTCAGGCCGAACATGCTGAG
BMP3	GCAGGGAGAGAGACCGAAG	TGGACCGTGCTGTACCTGT
BMP4	AAAGTCGCCGAGATTCAGGG	GACGGCACTCTTGCTAGGC
BMP5	AAGACTACGGAACCACGAAAGA	GGTGCAGAGGACGCTTGTTT
BMP6	AGCGACACCACAAAGAGTTCA	GCTGATGCTCCTGTAAGACTTGA
BMP7	TCGGCACCCATGTTCATGC	GAGGAAATGGCTATCTTGCAGG
BMP8a	CTGGTTGCTGAAGCGTCACAAG	AGTGACCACGAAAGGCTGTTGG
BMP8b	GGAGCCCCATTGGAAGGAG	CTCGGAGCGTCTGAAGATCC
BMP10	ACTGGTGCAAAGGGATCGTAT	AGTCTCCCACTCACTGTTGGT
BMP15	TGTGAACTCGTGCTTTTCATGG	CTCAATCAGGGGCAAAGTAGG
GAPDH	GGAGCGAGATCCCTCCAAAAT	GGCTGTTGTCATACTTCTCATGG

Table S2. Antibodies used in this study.

Antibody	Supplier	Cat no.	Application/Dilution
KINDLIN-2	Merck Millipore	MAB2617	WB (1:1000); IHC (1:200); IF (1:100)
KINDLIN-2	Proteintech	11453-1-AP	Co-IP (1:100); IHC (1:200); IF (1:100)
GAPDH	ZSGB-BIO	TA-08	WB (1:1000)
Runx2	HUABIO	ET1612-47	WB (1:1000); IHC (1:200); IF (1:100)
Flag	ZSGB-BIO	TA-5	WB (1:1000)
Osterix (SP7)	HUABIO	ER1914-47	WB (1:1000); IHC (1:200); IF (1:100)
CD31	BD Pharmingen	550274	WB (1:1000); IF (1:200)
Smad2/3	ABClonal	A18674	WB (1:1000); IF (1:100)
pSmad2/3	ABClonal	AP0548	WB (1:1000); IF (1:100)
TIE2	Invitrogen	14-5987-82	IF (1:200)
TIE2	ABClonal	A7222	WB (1:1000)
VE-Cadherin	Abcepta	AP73794	WB (1:1000); IF (1:100)
N-Cadherin	ABClonal	A19083	WB (1:1000); IF (1:100)
Smad1/5/9	Affinity	AF0614	WB (1:1000); IHC (1:200)
pSmad1/5/9	Affinity	AF8313	WB (1:1000); IHC (1:200)
Ubiquitin (Ub)	ABClonal	A19686	WB (1:1000)
Piezo1	ABClonal	A4340	WB (1:1000); IHC (1:200); Co-IP (1:100)
Piezo1	Proteintech	15939-1-AP	IF (1:200)

Osteocalcin (Ocn)	Bioss Antibodies	bs-4917R	WB (1:1000); IF (1:200)
TGFβ1	ABClonal	A21245	WB (1:1000); IF (1:200)
TGFβ2	ABClonal	RP00452	WB (1:1000); IF (1:200)
Tubulin	ZSGB-BIO	TA-10	WB (1:1000)
CyclinD1	ABClonal	A19038	IF (1:200)
NFATc1	ABClonal	A1539	WB (1:1000); IF (1:200)
Trim28	ABClonal	A19568	WB (1:1000); IF (1:100); Co-IP (1:100)

Table S3. Demographics of the osteoporosis patients involved in this study.

	Control	OP
Patients number	10	8
Gender	Female	Female
Age	64.21 ± 3.47	63.18 ± 4.54
Bone density (T-scores ±SD)	-0.37 ± 1.22	-2.83 ± 0.45

Table S4. GO analysis of differentially expressed genes after Kindlin-2 knockdown in HUVECs.

**Category**	**Term**	**Count**	**%**	**P-Value**
GOTERM_MF_DIRECT	protein binding	573	50.4	0.00046
GOTERM_MF_DIRECT	metal ion binding	132	11.6	0.025
GOTERM_MF_DIRECT	identical protein binding	95	8.4	0.0048
GOTERM_MF_DIRECT	DNA binding	75	6.6	0.009
GOTERM_MF_DIRECT	RNA polymerase II transcription factor activity	74	6.5	0.0052
GOTERM_MF_DIRECT	RNA polymerase II core promoter proximal region	64	5.6	0.046
GOTERM_MF_DIRECT	calcium ion binding	41	3.6	0.077
GOTERM_MF_DIRECT	protein heterodimerization activity	27	2.4	0.008
GOTERM_MF_DIRECT	protein kinase activity	24	2.1	0.051
GOTERM_MF_DIRECT	structural constituent of chromatin	21	1.8	4.5E-09
GOTERM_MF_DIRECT	signaling receptor activity	16	1.4	0.065
GOTERM_MF_DIRECT	serine-type endopeptidase activity	15	1.3	0.034
GOTERM_MF_DIRECT	extracellular matrix structural constituent	12	1.1	0.032
GOTERM_MF_DIRECT	integrin binding	12	1.1	0.079
GOTERM_MF_DIRECT	collagen binding	9	0.8	0.0076
GOTERM_MF_DIRECT	peptidase activity	9	0.8	0.066
GOTERM_MF_DIRECT	kinase binding	9	0.8	0.066
GOTERM_MF_DIRECT	double-stranded RNA binding	8	0.7	0.038
GOTERM_MF_DIRECT	insulin-like growth factor binding	7	0.6	0.0003
GOTERM_MF_DIRECT	protein kinase activator activity	6	0.5	0.019
GOTERM_MF_DIRECT	endoribonuclease activity	6	0.5	0.019

Table S5. Mass spectrometry identified E3 ubiquitin ligases in the interactome of Kindlin-2.

Gene	ΣCoverage	Σ# Unique Peptides	Σ# Peptides
Trim28	1.27	1	1

Supplementary Figures

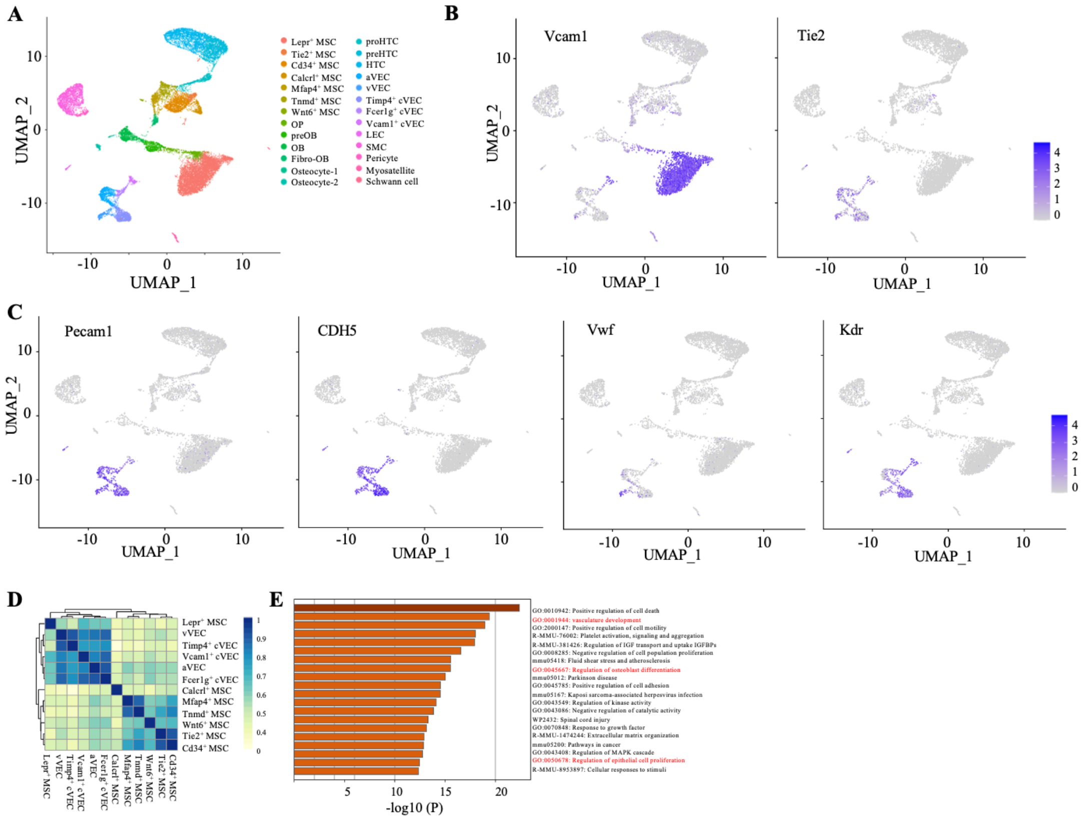

Figure S1. Characterization of cell types in BMSC pools.

(**A**) Visualization of cell types in UMAP. (**B-C**) Expression levels of endothelial marker genes *Vcam1*, *Tie2* (**B**), *Pecam1*, *CDH5*, *Vwf* and *Kdr* (**C**). (**D**) Heat map of highly expressed genes in all cell types. (**E**) Enrichment heatmap of genes co-expressed in Lepr^{+} MSC and VEC.

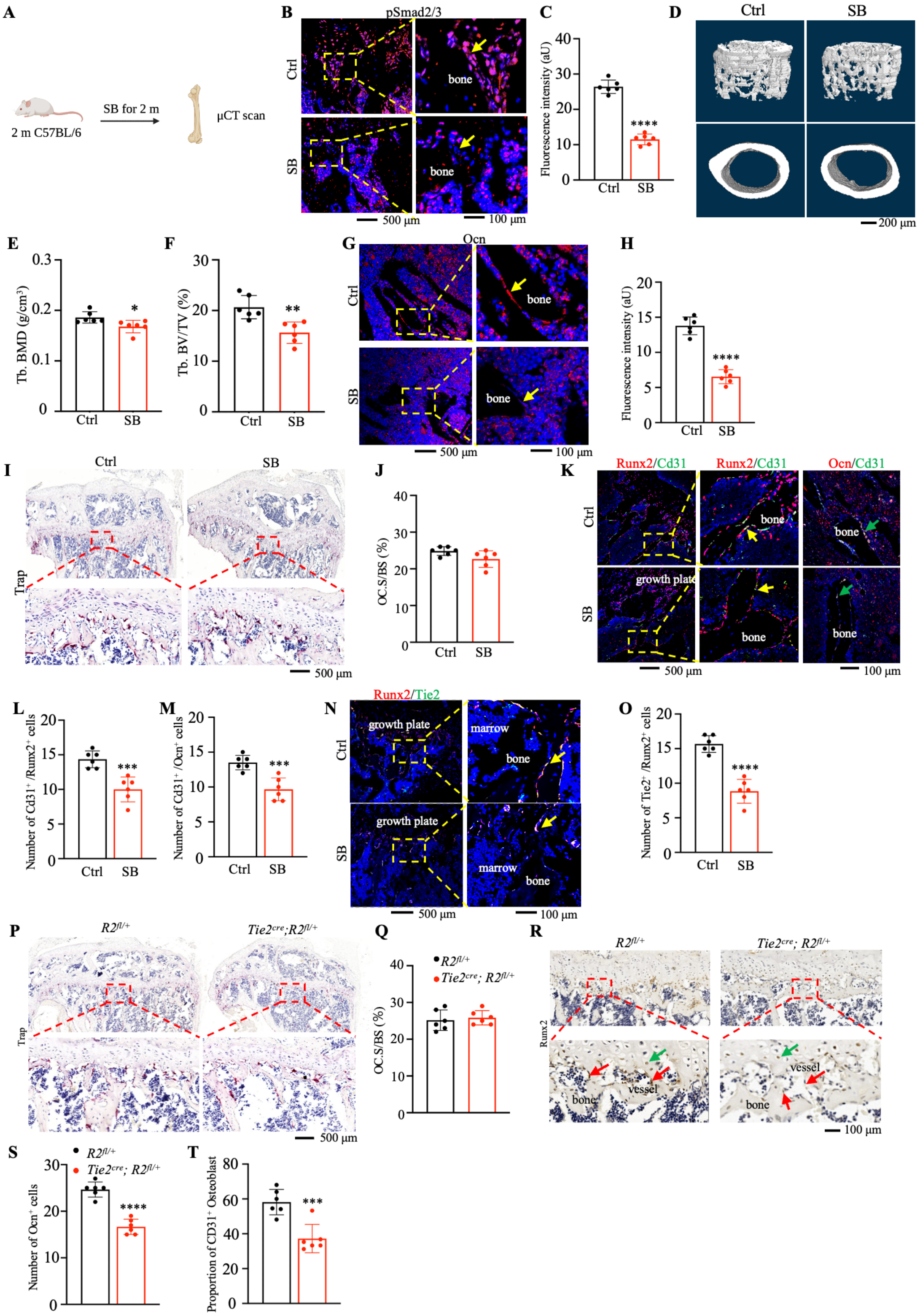

Figure S2. Inhibition of EC-to-OSB decrease bone mass.

(**A**) Schematic diagram of SB administration to mice. (**B-C**) IF staining. Tibial sections of C57/BL6 mice after being treated with SB for 2 months were subjected to IF staining using indicated antibodies. Scale bar, 500 μm and 100 μm. The yellow arrow indicated

IF signal of osteoblasts on trabecular bone surface. (**D-F**) Three-dimensional (3D) reconstruction (**D**) and quantitative analyses of BMD (**E**), BV/TV (**F**) from µCT scans of the distal femurs of C57/BL6 mice after intraperitoneally administration of SB for 2 months. Scale bar, 200 µm. Female mice, N = 6 mice for each group. IF staining if Ocn (**G**) and its quantitative analyses (**H**). Tibial sections of C57/BL6 mice after being treated with SB for 2 months were subjected to IF staining using indicated antibodies. Scale bar, 300 µm and 100 µm. The yellow arrows indicated IF signals of osteoblasts on trabecular bone surface. N = 6 mice for each group. (**I-J**) Trap staining of tibial sections from C57/BL6 mice after being treated with SB for 2 months. Scale bar, 500 µm. (**K-O**) IF staining. Tibial sections from C57/BL6 mice after administration of SB for 2 months were subjected to IF staining using antibodies against Runx2, Ocn and Cd31. Female mice, N = 6 mice for each group. The yellow arrow indicated $Runx2^+$ $Cd31^+$ cells, while the green arrow indicated Ocn^+ $Cd31^+$ cells. Scale bar, 500 µm, 100 µm, 100 µm. (**P-Q**) Trap staining of tibial sections from $Tie2^{cre}$; $Runx2^{fl/+}$ mice and control mice at 3 month of age. Scale bar, 500 µm. (**R**) IHC staining. Tibial sections of $Tie2^{cre}$; $Runx2^{fl/+}$ mice and control mice at 3 month of age were subjected to IHC staining using indicated antibodies. Scale bar, 100 µm. The red arrow indicated endothelial cells and the blue arrow indicated hypertrophic chondrocytes. (**P-Q**) Statistics on the total number of osteoblasts and the proportion of CD31 positive osteoblasts. $^{*}P < 0.05$, $^{**}P < 0.01$, $^{***}P < 0.001$, $^{****}P < 0.0001$, versus controls. Student's *t* test. Results are expressed as the mean ± sd.

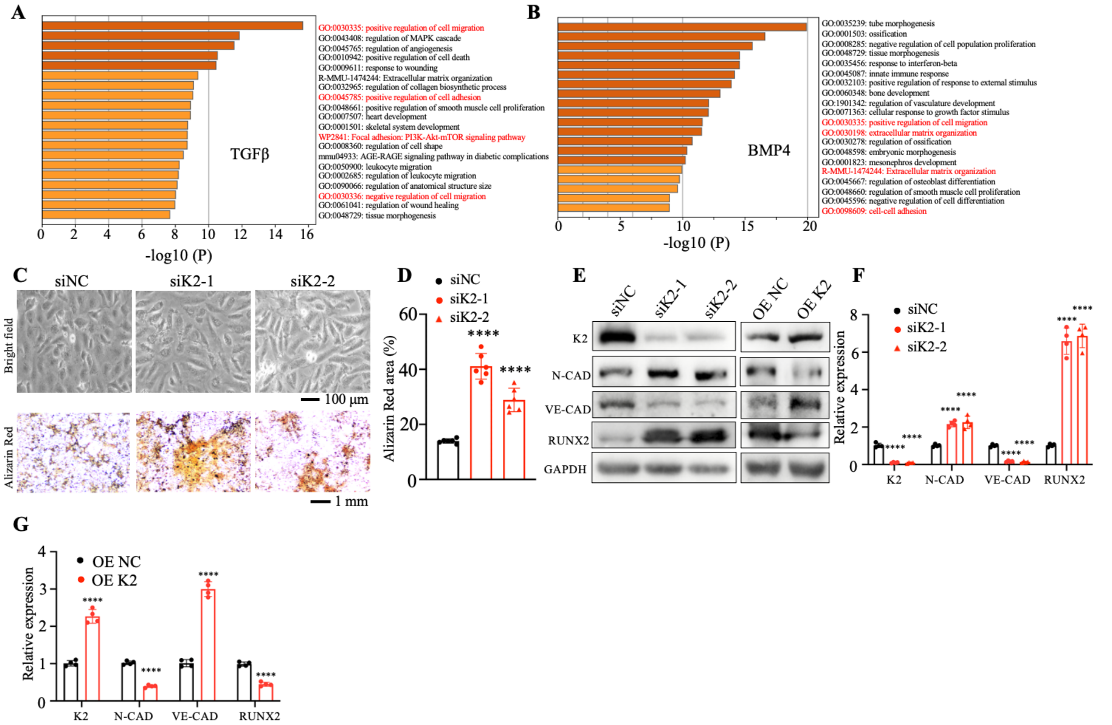

Figure S3. Kindlin-2 respond to BMP4 and TGFβ stimulation.

(**A-B**) Enrichment heatmap of differentially expressed genes after TGFβ (**A**) and BMP4 (**B**) treatment in endothelial cells. (**C-D**) Bright field, Alizarin Red staining (**C**) and its quantitative analyses (**D**) of HUVECs after KINDLIN-2 was knocked down. Scale bar, 100 μm and 1 mm. (**E-G**) WB and its quantitative analyses. Protein extracts were isolated from HUVECs after knockdown or overexpression of KINDLIN-2. N = 4 biologically independent experiments. $^{*}P < 0.05$, $^{**}P < 0.01$, $^{***}P < 0.001$, $^{****}P < 0.0001$, versus controls. Student's *t* test. Results are expressed as the mean ± sd.

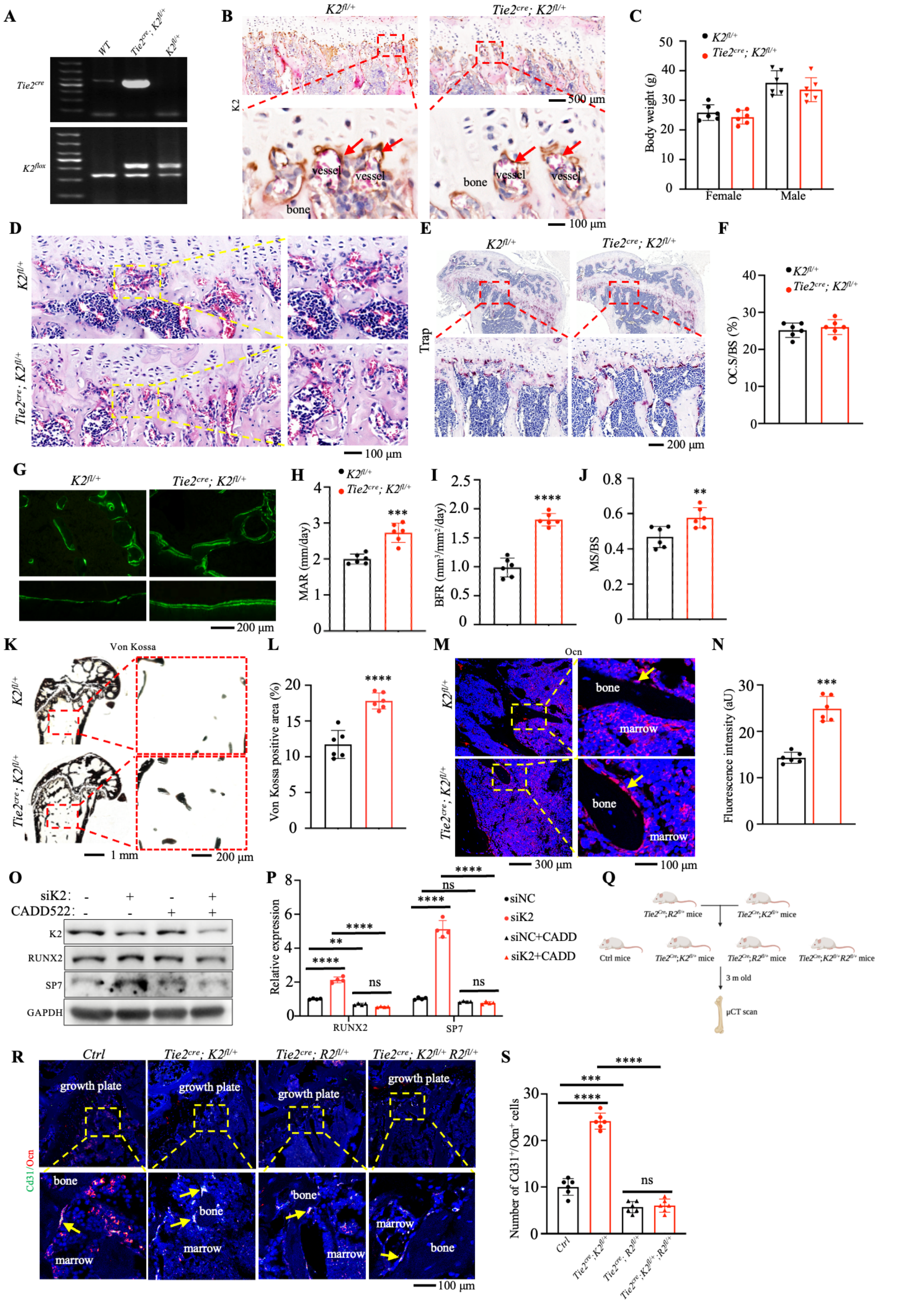

Figure S4. Endothelial Kindlin-2 haploinsufficiency mice have increased EC-to-OSB.

(**A**) Genotyping. (**B**) IHC staining of tibial sections from *Tie2^{cre}; K2$^{fl/+}$* mice and control mice at age of 2 months. The red arrow indicated endothelial cells. (**C**) Body weight of

Tie2^{cre}*; K2*$^{fl/+}$ mice and control mice at age of 2 months. (**D**) H&E staining of bone sections from *Tie2*^{cre}*; K2*$^{fl/+}$ mice and control mice at age of 2 months. (**E**) Trap staining and its quantitative analyses (**F**) of bone sections from *Tie2*^{cre}*; K2*$^{fl/+}$ mice and control mice at age of 2 months. Female mice, N = 6 mice for each group. Scale bar, 200 μm. (**G-J**) Calcein double labeling (**G**) and quantitative analyses of the mineral apposition rate (MAR) (**H**), bone formation rate (BFR) (**I**) and mineralizing surface per bone surface (MS/BS) (**J**). Scale bar, 200 μm. N = 6 mice for each group. (**K-L**) Von Kossa staining (**K**) and quantitative analyses (**L**) of nondemineralized bone sections from *Tie2*^{cre}*; K2*$^{fl/+}$ mice and control mice at 2 month of age. Scale bar, 1 mm and 200 μm. N = 6 mice for each group. (**M-N**) IF staining if Ocn (**M**) and its quantitative analyses (**N**). Tibial sections of *Tie2*^{cre}*; K2*$^{fl/+}$ and control mice at age of 2 months were subjected to IF staining using indicated antibodies. Scale bar, 300 μm and 100 μm. The yellow arrows indicated IF signals of osteoblasts on trabecular bone surface. Female mice, N = 6 mice for each group. (**O-P**) WB and quantitative analyses of HUVECs after being treated with RUNX2 inhibitor CADD522 and KINDLIN-2 knockdown. (**Q**) Schematic diagram of to *Tie2*^{cre}*;K2*$^{fl/+}$*; Runx2*$^{fl/fl}$ mice. (**R-S**) IF staining of endothelial derived osteoblasts and its quantitative analyses. Tibial sections of *Tie2*^{cre}*; K2*$^{fl/+}$*; Runx2*$^{fl/+}$ and control mice were subjected to IF staining using indicated antibodies. The yellow arrow indicated $Cd31^+/Ocn^+$ cells. Scale bar, 100 μm. $^*P < 0.05$, $^{**}P < 0.01$, $^{***}P < 0.001$, $^{****}P < 0.0001$, versus controls. Student's *t* test or one-way ANOVA. Results are expressed as the mean ± sd.

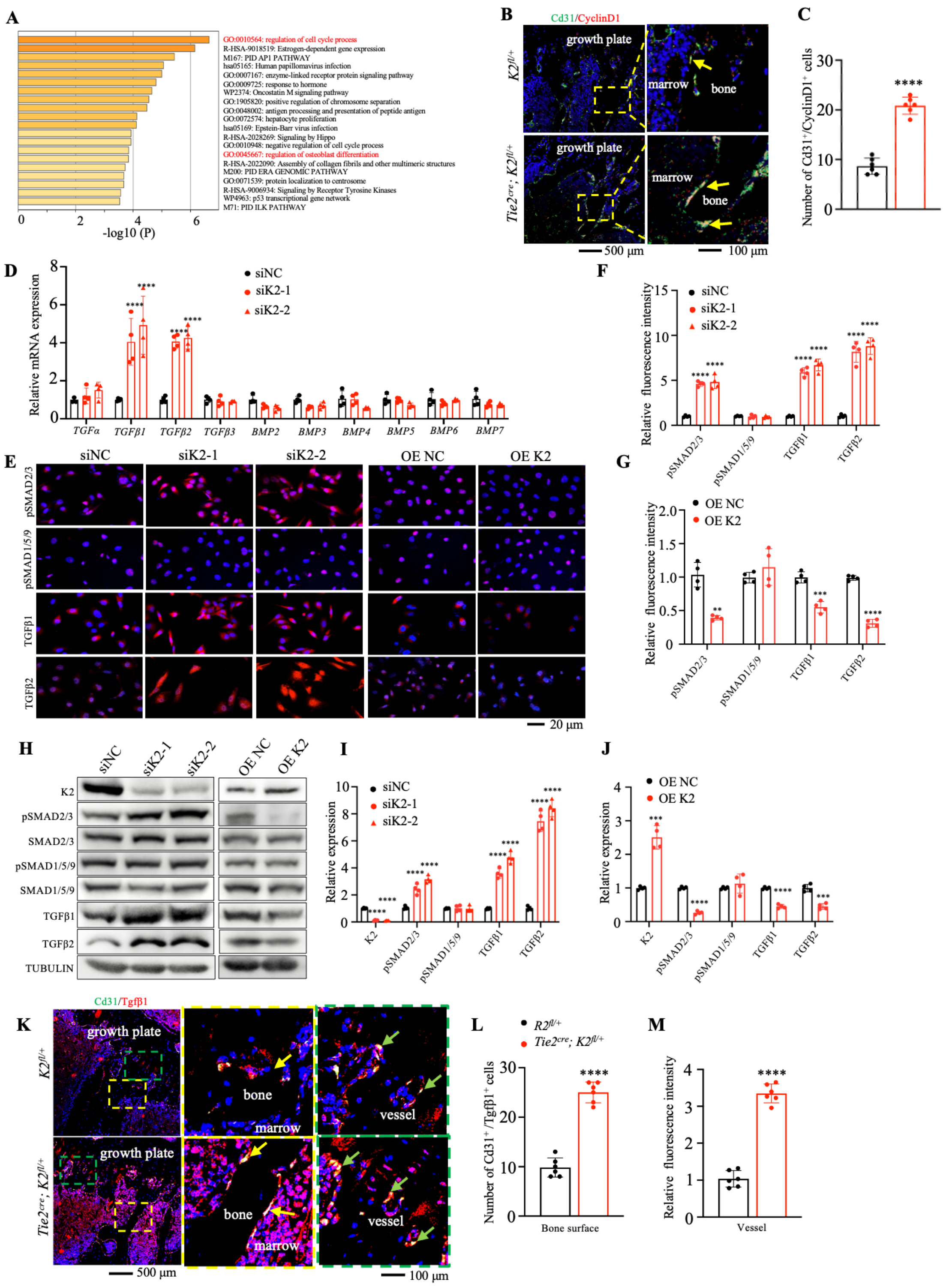

Figure S5. Endothelial Kindlin-2 haploinsufficiency results in an increased TGFβ expression and cell proliferation.

(**A**) Enrichment heatmap of differentially expressed genes after KINDLIN-2 knockdown in HUVECs. (**B-C**) IF staining and quantitative analyses. Tibial sections of *Tie2^{cre}; K2$^{fl/+}$* and control mice were subjected to IF staining using indicated antibodies.

Scale bar, 500 μm and 100 μm. The yellow arrow indicates $Cd31^+$ osteoblasts. Scale bar, 100 μm. (**D**) qRT-PCR analyses using RNA samples isolated from HUVECs after KINDLIN-2 knockdown. (**E-G**) IF staining and quantitative analyses of HUVECs after KINDLIN-2 was knocked down or over expressed. N = 4 biologically independent experiments. Scale bar, 20 μm. (**H-J**) WB analyses (**H**) and and its quantitative analyses (**I-J**) of HUVECs after KINDLIN-2 was knocked down or over expressed. N = 4 biologically independent experiments. (**K-M**) IF staining. Tibial sections of $Tie2^{cre}$; $K2^{fl/+}$ and control mice were subjected to IF staining using indicated antibodies. Scale bar, 500 μm and 100 μm. The yellow arrow indicated $Cd31^+$ osteoblasts. $^{*}P < 0.05$, $^{**}P < 0.01$, $^{***}P < 0.001$, $^{****}P < 0.0001$, versus controls. Student's *t* test or one-way ANOVA. Results are expressed as the mean ± sd.

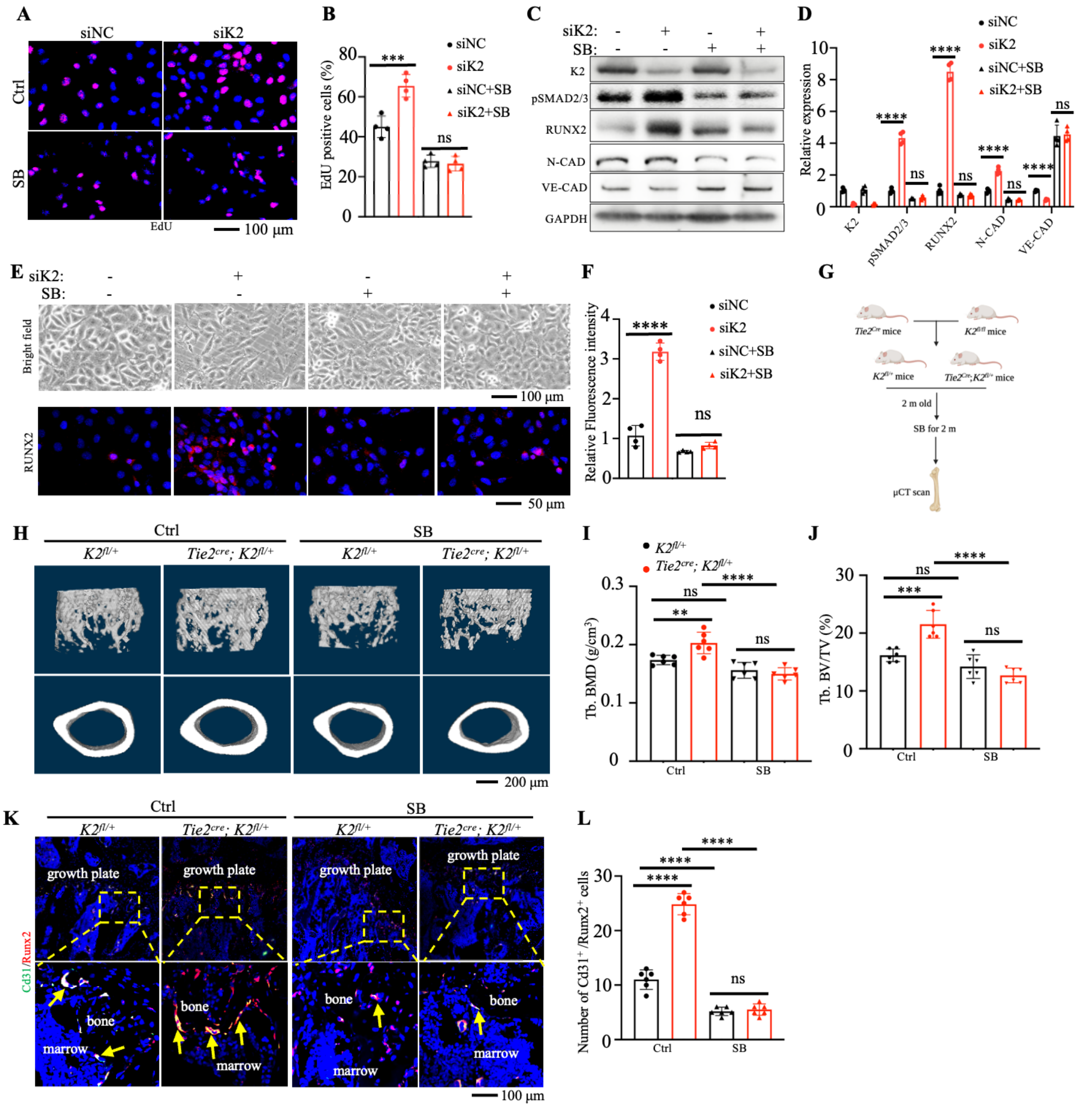

Figure S6. Endothelial Kindlin-2 deficiency increases EC-to-OSB through enhanced TGFβ pathway.

(**A-B**) EdU staining and quantitative analyses of HUVECs after KINDLIN-2 knockdown and SB treatment. Scale bar, 100 μm. (**C-D**) WB (**C**) and its quantitative analyses (**D**) of HUVECs after KINDLIN-2 was knocked down with or without SB treatment . N = 4 biologically independent experiments. (**E-F**) Bright field, IF staining (**E**) and its quantitative analyses (**F**) of HUVECs after KINDLIN-2 was knocked down with or without SB treatment. Scale bar, 100 μm and 50 μm. N = 4 biologically independent experiments. (**G**) Schematic diagram of SB administration to mice. (**H-J**) 3D reconstruction (**H**) and quantitative analyses of BMD (**I**), BV/TV (**J**) from μCT

scans of the distal femurs of *Tie2*^{cre}*; K2*$^{fl/+}$ mice and control mice with or without SB treatment. Scale bar, 200 μm. N = 6 mice for each group. (**K-L**) IF staining of endothelial derived osteoblasts and its quantitative analyses. Tibial sections of *Tie2*^{cre}*; K2*$^{fl/+}$ mice and control mice with or without SB treatment were subjected to IF staining with indicated antibodies. The yellow arrow indicated Cd31^{+}/Runx2^{+} cells. *P < 0.05, **P < 0.01, ***P < 0.001, ****P < 0.0001, versus controls. Student's *t* test or one-way ANOVA. Results are expressed as the mean ± sd.

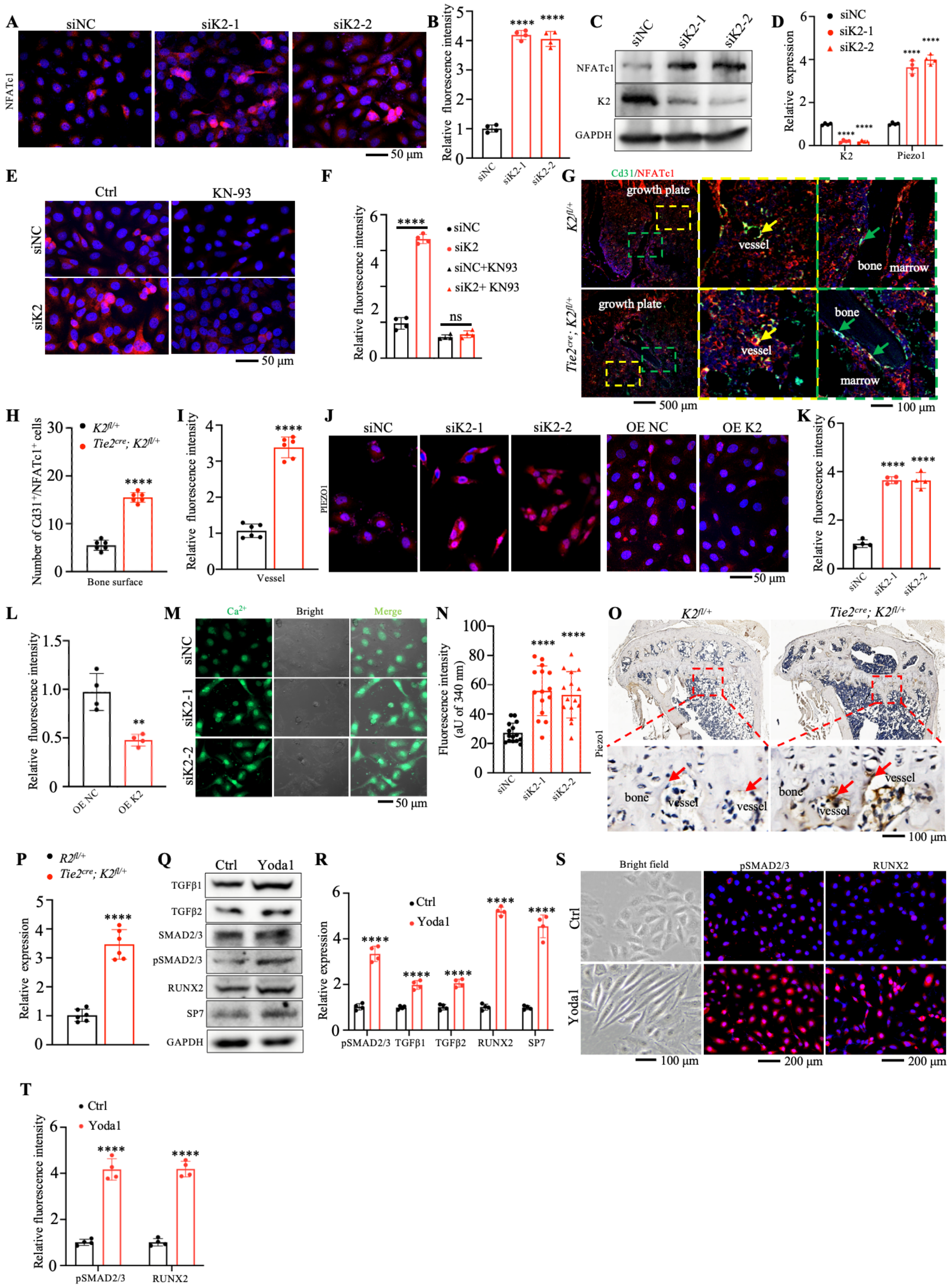

Figure S7. Endothelial Piezo1 regulates EC-to-OSB.

(**A-D**) IF staining (**A-B**) and WB analyses (**C-D**) of HUVECs after KINDLIN-2 knockdown. Scale bar, 50 μm. N = 4 biologically independent experiments. (**E-F**) IF staining. HUVECs after KINDLIN-2 knockdown and KN-93 treatment were subjected to IF staining using indicated antibodies. Scale bar, 50 μm. N = 4 biologically

independent experiments. (**G-I**) IF staining and quantitative analyses. Tibial sections of *Tie2*^{cre}*; K2*$^{fl/+}$ and control mice at age of 2 months were subjected to IF staining using indicated antibodies. Scale bar, 500 μm and 100 μm. Female mice, N = 6 mice per group. The yellow arrows indicated endothelial cells, and the green arrows indicated osteoblasts. (**J-L)** IF staining and quantitative analyses. HUVECs after KINDLIN-2 overexpression were subjected to IF staining for PIEZO1. Scale bar, 50 μm. N = 4 biologically independent experiments. (**M-N**) IF staining and quantitative analyses of Ca^{2+} by Furo-2 in HUVECs. Scale bar, 50 μm. (**O-P**) IHC staining and quantitative analyses of tibial sections of *Tie2*^{cre}*; K2*$^{fl/+}$ and control mice at 2 month of age. Female mice, N = 6 mice for each group. The red arrow indicated endothelial cells. Scale bar, 100 μm. (**Q-R**) WB and quantitative analyses of HUVECs after being treated with Yoda1. N = 4 biologically independent experiments. (**S-T**) Bright field, IF staining and quantitative analyses of HUVECs after being treated with Yoda1. Scale bar, 100 μm and 200 μm. N = 4 biologically independent experiments. $^{*}P < 0.05$, $^{**}P < 0.01$, $^{***}P < 0.001$, $^{****}P < 0.0001$, versus controls. Student's *t* test or one-way ANOVA. Results are expressed as the mean ± sd.

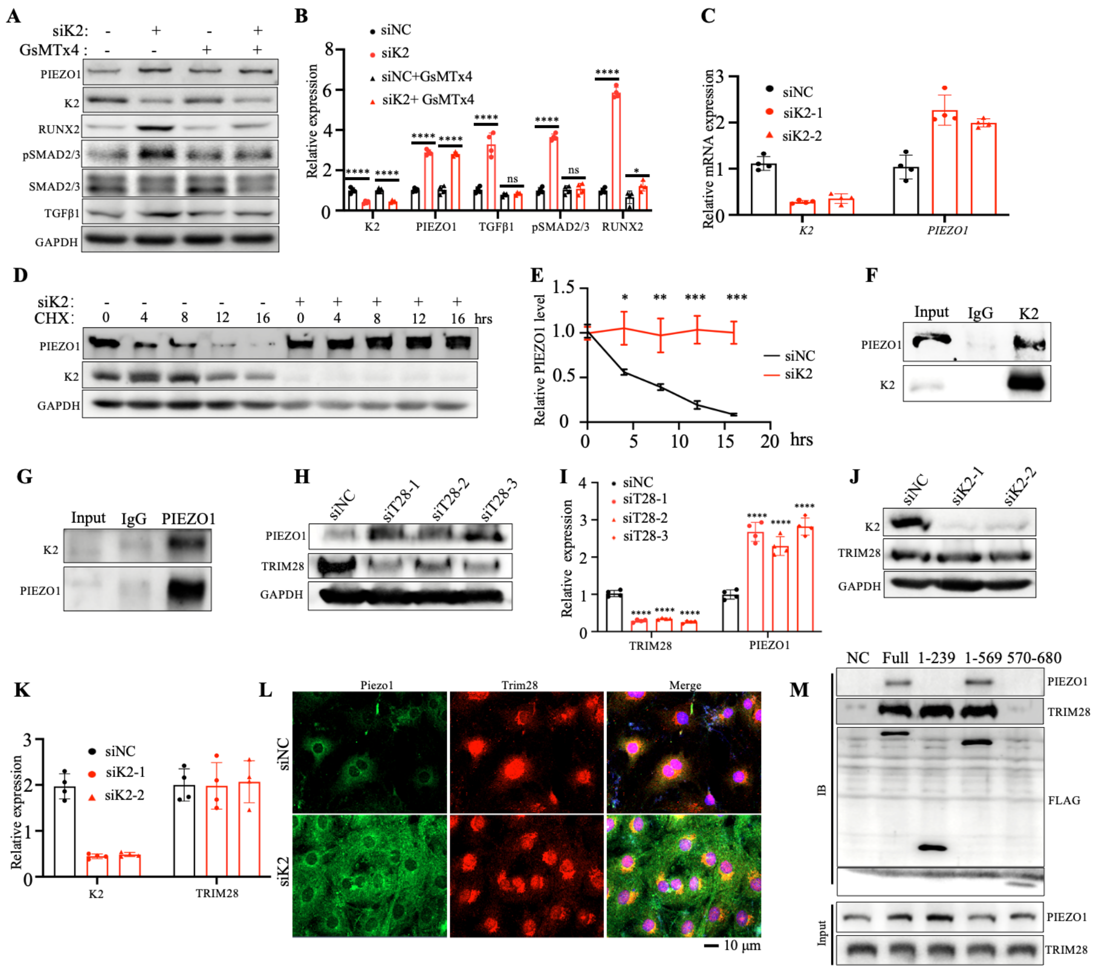

Figure S8. Kindlin-2 knockdown increases Piezo1 protein stability.

(**A-B**)WB and quantitative analyses of of HUVECs with KINDLIN-2 knockdown followed by GsMTx4 treatment. Scale bar, 50 μm. (**C**) qRT-PCR analyses. RNA samples isolated from HUVECs after KINDLIN-2 knockdown were used for qRT-PCR analyses. (**D-E**) CHX assays and its quantitative analyses. (**F-G**) Co-IP assays. Cell lysates from HUVECs were used for IP and IB assays for detecting the endogenous interaction between KINDLIN-2 and PIEZO1. (**H-I**) WB and quantitative analyses. Protein extracts were isolated from HUVECs after TRIM28 knockdown. N = 4 biologically independent experiments. (**J-K**) WB analyses. Protein extracts were isolated from HUVECs after KINDLIN-2 knockdown. N = 4 biologically independent experiments. (**L**) IF staining for detecting the colocalization of KINDLIN-2 and PIEZO1 in HUVECs after TRIM28 knockdown. Scale bar, 10 μm. (**M**) Co-IP assays. HUVECs were co-transfected with plasmid constructs for expression of full-length

Kindlin-2 and its truncations (with Flag tag). 48 hours later, cell lysates were prepared and used for Co-IP assays. $^{*}P < 0.05$, $^{**}P < 0.01$, $^{***}P < 0.001$, $^{****}P < 0.0001$, versus controls. Student's *t* test or one-way ANOVA. Results are expressed as the mean ± sd.

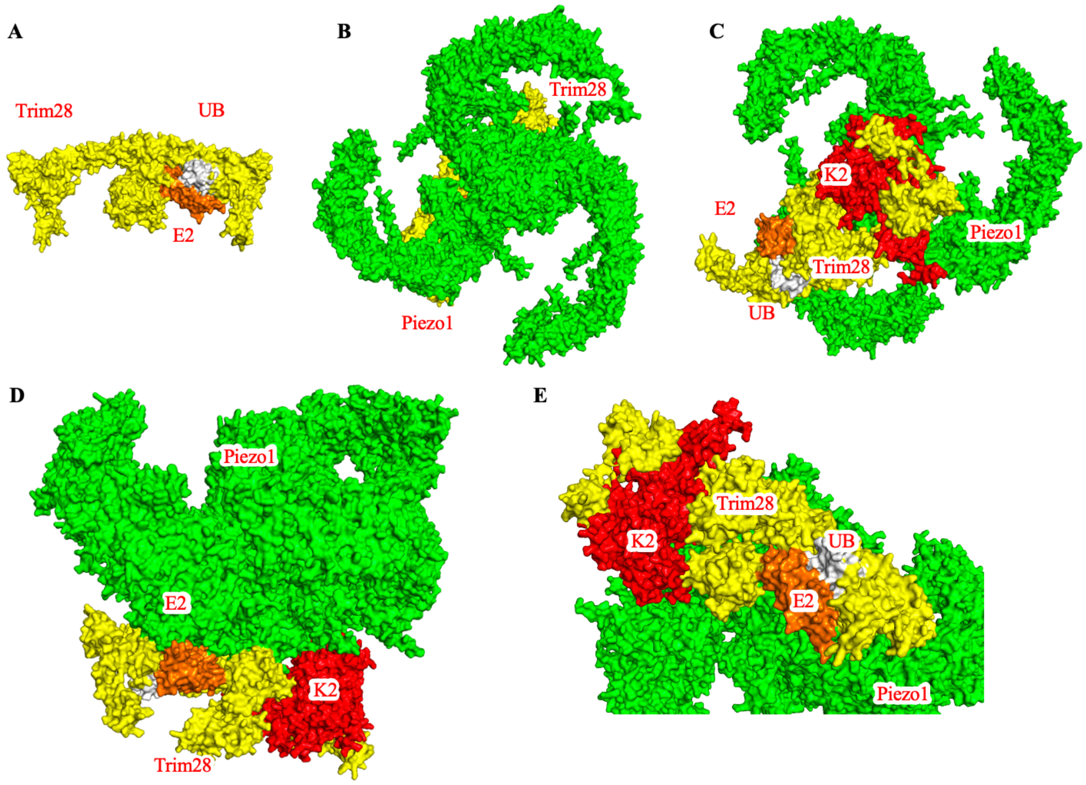

Figure S9. Predicted protein structure of the Kindlin-2/Trim28/Piezo1 complex. (**A**) Protein structure of the Trim28/E2/UB complex, photographs were displayed by Pymol and Chimera. UBs were showed in gray, E2 in orange, and Trim28 in yellow. (**B-E**) Protein structure of the Kindlin-2/Trim28/Piezo1 complex. Structure showed in Top view (**B**), bottom view (**C**), side view (**D**) and zoomed side view (**E**), photographs were displayed by Pymol and Chimera. UBs were showed in gray, E2 in orange, Trim28 in yellow, Kindlin-2 in red, and Piezo1 in green.

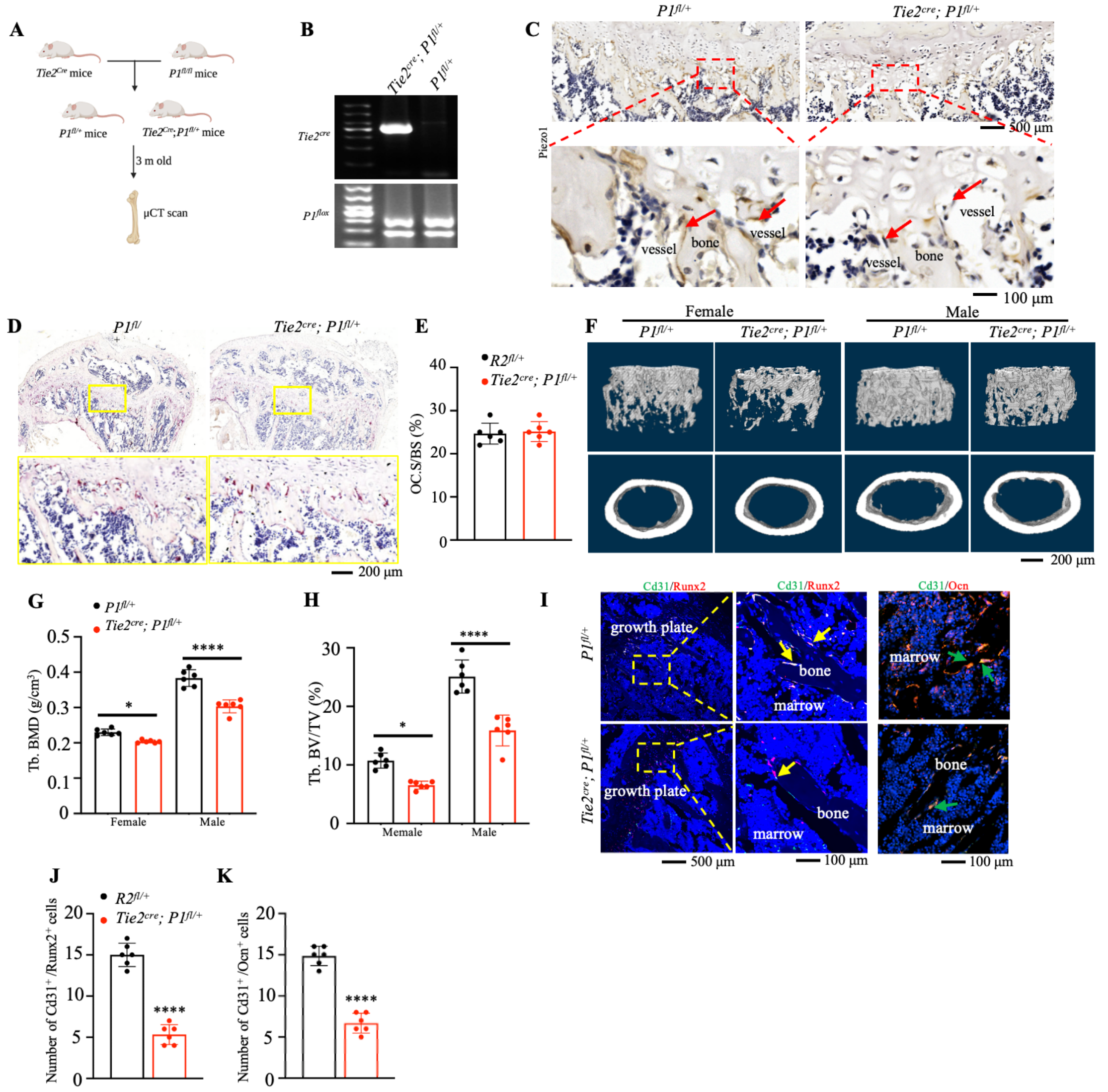

Figure S10. Endothelial Piezo1 haploinsufficiency decrease bone mass.

A) Schematic diagram of generating *Tie2^cre^; Piezo1^fl/+^* mice. (**B**) Genotyping of *Tie2^cre^; Piezo1^fl/+^* mice. (**C**) IHC staining of tibial sections from *Tie2^cre^; Piezo1^fl/+^* mice and control mice at 3 month of age. The red arrow indicated endothelial cells. Scale bar, 100 µm. (**D-E**) Trap staining of tibial sections from *Tie2^cre^; Peizo1^fl/+^* and control mice at age of 3 months. Scale bar, 200 µm. Female mice, N = 6 mice per group. (**F-H**) 3D reconstruction (**F**) and quantitative analyses of BMD (**G**), BV/TV (**H**) from µCT scans of the distal femurs of *Tie2^cre^; Piezo1^fl/+^* mice and control mice. N = 6 mice for each group. Scale bar, 200 µm. (**I-K**) IF staining and quantitative analyses. Tibial sections of *Tie2^cre^; Piezo1^fl/+^* and control mice at age of 3 months were subjected to IF staining

using indicated antibodies. Scale bar, 500 μm and 100 μm. Female mice, N = 6 mice per group. The yellow arrows and the green arrows indicated endothelial cells.

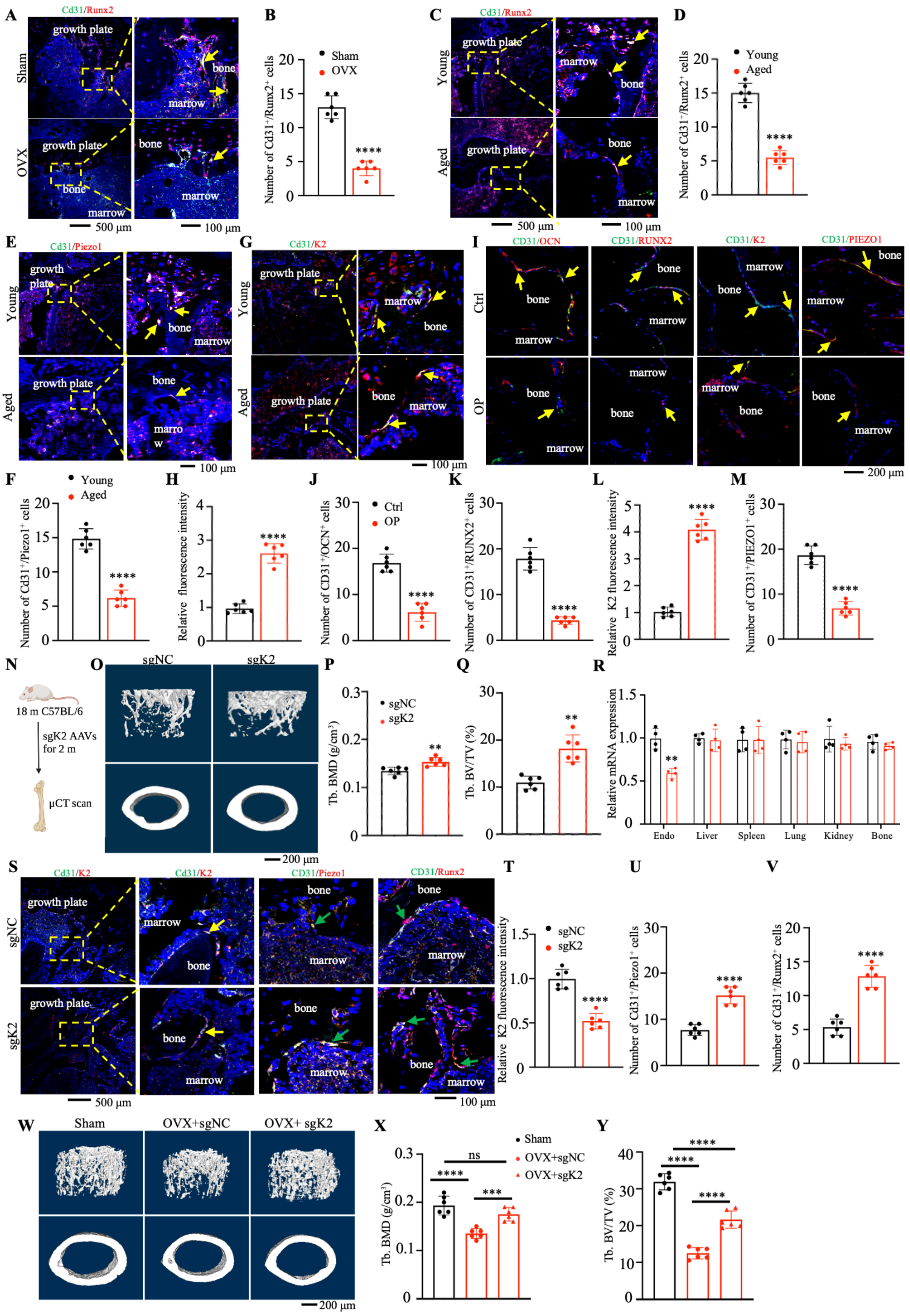

Figure S11. EC-to-OSB mediated bone homeostasis is related to osteoporosis in humans.

(**A-D**) IF staining and quantitative analyses. Tibial sections of OVX mice (**A-B**) and aged mice (**C-D**) were subjected to IF staining using indicated antibodies. Scale bar,

500 μm and 100 μm. Female mice, N = 6 mice per group. The yellow arrows and the green arrows indicated endothelial cells. (**E-H**) IF staining of endothelial derived osteoblasts and its quantitative analyses. Tibial sections from young and aged mice were subjected to IF staining using antibodies against Cd31 and Piezo1 (**E-F**) and Kindlin-2 (**G-H**). N = 6 mice for each group. The yellow arrow indicated $Cd31^+$ cells. Scale bar, 100 μm. (**I-M**) IF staining of endothelial derived osteoblasts and its quantitative analyses. Bone sections from OP patients and normal controls were subjected to IF staining using indicated antibodies. The yellow arrow indicates $CD31^+$ cells. Scale bar, 200 μm. (**N**) Schematic diagram of endothelial *Kindlin-2* mRNA editing administration to mice. (**O-Q**) 3D reconstruction (**O**) and quantitative analyses of BMD (**P**), BV/TV (**Q**) from μCT scans of the distal femurs of sgK2 treated mice and control mice. Female mice, N = 6 mice for each group. Scale bar, 200 μm. (**R**) qRT-PCR analyses using RNA samples isolated from endothelial cells or organs after K AAVs injection for 2 months. (**S-V**) IF staining of endothelial derived osteoblasts and its quantitative analyses. Tibial sections of sgK2 treated mice and control mice were subjected to IF staining using indicated antibodies. The yellow arrow indicated $Cd31^+/Runx2^+$ cells on trabecular surface. Scale bar, 100 μm. (**W-Y**) 3D reconstruction (**W**) and quantitative analyses of BMD (**X**), BV/TV (**Y**) from μCT scans of the distal femurs of sgK2 treated OVX mice, OVX mice and control mice. Female mice, N = 6 mice for each group. Scale bar, 200 μm.$^{*}P < 0.05$, $^{**}P < 0.01$, $^{***}P < 0.001$, $^{****}P < 0.0001$, versus controls. Student's *t* test. Results are expressed as the mean ± standard deviation (sd).

References

Aikawa, E., Nahrendorf, M., Figueiredo, J.L., Swirski, F.K., Shtatland, T., Kohler, R.H., Jaffer, F.A., Aikawa, M., and Weissleder, R. (2007). Osteogenesis associates with inflammation in early-stage atherosclerosis evaluated by molecular imaging in vivo. Circulation 116, 2841-2850.

Becht, E., McInnes, L., Healy, J., Dutertre, C.A., Kwok, I.W.H., Ng, L.G., Ginhoux, F., and Newell, E.W. (2018). Dimensionality reduction for visualizing single-cell data using UMAP. Nat Biotechnol.

Bouxsein, M.L., Boyd, S.K., Christiansen, B.A., Guldberg, R.E., Jepsen, K.J., and Müller, R. (2010). Guidelines for assessment of bone microstructure in rodents using micro–computed tomography. Journal of bone and mineral research 25, 1468-1486.

Cahalan, S.M., Lukacs, V., Ranade, S.S., Chien, S., Bandell, M., and Patapoutian, A. (2015). Piezo1 links mechanical forces to red blood cell volume. Elife 4.

Cummings, S.R., San Martin, J., McClung, M.R., Siris, E.S., Eastell, R., Reid, I.R., Delmas, P., Zoog, H.B., Austin, M., Wang, A., *et al.* (2009). Denosumab for prevention of fractures in postmenopausal women with osteoporosis. N Engl J Med 361, 756-765.

Fu, X., Zhou, B., Yan, Q., Tao, C., Qin, L., Wu, X., Lin, S., Chen, S., Lai, Y., Zou, X., *et al.* (2020). Kindlin-2 regulates skeletal homeostasis by modulating PTH1R in mice. Signal Transduction and Targeted Therapy 5.

Gao, H., Zhou, L., Zhong, Y., Ding, Z., Lin, S., Hou, X., Zhou, X., Shao, J., Yang, F., Zou, X., *et al.* (2022). Kindlin-2 haploinsufficiency protects against fatty liver by targeting Foxo1 in mice. Nat Commun 13, 1025.

Guan, J., Wang, G., Wang, J., Zhang, Z., Fu, Y., Cheng, L., Meng, G., Lyu, Y., Zhu, J., Li, Y., *et al.* (2022). Chemical reprogramming of human somatic cells to pluripotent stem cells. Nature.

Jumper, J., Evans, R., Pritzel, A., Green, T., Figurnov, M., Ronneberger, O., Tunyasuvunakool, K., Bates, R., Zidek, A., Potapenko, A., *et al.* (2021). Highly accurate protein structure prediction with AlphaFold. Nature.

Kisanuki, Y.Y., Hammer, R.E., Miyazaki, J., Williams, S.C., Richardson, J.A., and Yanagisawa, M. (2001). Tie2-Cre transgenic mice: a new model for endothelial cell-lineage analysis in vivo. Dev Biol 230, 230-242.

Li, X., Zhang, B., Wu, Q., Ci, X., Zhao, R., Zhang, Z., Xia, S., Su, D., Chen, J., Ma, G., *et al.* (2015). Interruption of KLF5 acetylation converts its function from tumor suppressor to tumor promoter in prostate cancer cells. Int J Cancer 136, 536-546.

Liang, M.P., Banatao, D.R., Klein, T.E., Brutlag, D.L., and Altman, R.B. (2003). WebFEATURE: An interactive web tool for identifying and visualizing functional sites on macromolecular structures. Nucleic Acids Res 31, 3324-3327.

Lin, S.C., Lee, Y.C., Yu, G., Cheng, C.J., Zhou, X., Chu, K., Murshed, M., Le, N.T., Baseler, L., Abe, J.I., *et al.* (2017). Endothelial-to-Osteoblast Conversion Generates Osteoblastic Metastasis of Prostate Cancer. Dev Cell 41, 467-480 e463.

Medici, D., Shore, E.M., Lounev, V.Y., Kaplan, F.S., Kalluri, R., and Olsen, B.R. (2010). Conversion of vascular endothelial cells into multipotent stem-like cells. Nat Med 16, 1400-1406.

Pettersen, E.F., Goddard, T.D., Huang, C.C., Couch, G.S., Greenblatt, D.M., Meng, E.C., and Ferrin, T.E. (2004). UCSF Chimera—a visualization system for exploratory research and analysis. Journal

of computational chemistry 25, 1605-1612.
Takarada, T., Hinoi, E., Nakazato, R., Ochi, H., Xu, C., Tsuchikane, A., Takeda, S., Karsenty, G., Abe, T., and Kiyonari, H. (2013). An analysis of skeletal development in osteoblast‐specific and chondrocyte-specific runt-related transcription factor-2 (Runx2) knockout mice. Journal of Bone and Mineral Research 28, 2064-2069.
Tang, W., Ding, Z., Gao, H., Yan, Q., Liu, J., Han, Y., Hou, X., Liu, Z., Chen, L., Yang, D., *et al.* (2023). Targeting Kindlin-2 in adipocytes increases bone mass through inhibiting FAS/PPARγ/FABP4 signaling in mice. Acta Pharmaceutica Sinica B.
Waterhouse, A., Bertoni, M., Bienert, S., Studer, G., Tauriello, G., Gumienny, R., Heer, F.T., de Beer, T.A.P., Rempfer, C., Bordoli, L., *et al.* (2018). SWISS-MODEL: homology modelling of protein structures and complexes. Nucleic Acids Res 46, W296-W303.
Wu, C., Jiao, H., Lai, Y., Zheng, W., Chen, K., Qu, H., Deng, W., Song, P., Zhu, K., Cao, H., *et al.* (2015). Kindlin-2 controls TGF-beta signalling and Sox9 expression to regulate chondrogenesis. Nat Commun 6, 7531.
Yan, Q., Gao, H., Yao, Q., Ling, K., and Xiao, G. (2022). Loss of phosphatidylinositol-4-phosphate 5-kinase type-1 gamma (Pip5k1c) in mesenchymal stem cells leads to osteopenia by impairing bone remodeling. J Biol Chem 298, 101639.
Yan, Y., Zhang, D., Zhou, P., Li, B., and Huang, S.Y. (2017). HDOCK: a web server for protein-protein and protein-DNA/RNA docking based on a hybrid strategy. Nucleic Acids Res 45, W365-W373.
Yuan, S., Chan, H.C.S., Filipek, S., and Vogel, H. (2016). PyMOL and Inkscape Bridge the Data and the Data Visualization. Structure 24, 2041-2042.
Zhou, Y., Zhou, B., Pache, L., Chang, M., Khodabakhshi, A.H., Tanaseichuk, O., Benner, C., and Chanda, S.K. (2019). Metascape provides a biologist-oriented resource for the analysis of systems-level datasets. Nat Commun 10, 1523.
Zhu, K., Lai, Y., Cao, H., Bai, X., Liu, C., Yan, Q., Ma, L., Chen, D., Kanaporis, G., Wang, J., *et al.* (2020). Kindlin-2 modulates MafA and β-catenin expression to regulate β-cell function and mass in mice. Nature Communications 11, 484.
